# Determinants of Voluntary Counseling and Testing Service Uptake Among Adult Sub-Saharan Africans: A Systematic Review and Meta-Analysis

**DOI:** 10.3389/phrs.2022.1604065

**Published:** 2022-08-03

**Authors:** Muhammed Lamin Sambou, Juncheng Dai, Xiaoyu Zhao, Tongtong Hong, Til Bahadur Basnet, Gifty Marley, Alima Sambou, El Hafa Fadoua, Muhammad Naveed

**Affiliations:** School of Public Health, Nanjing Medical University, Nanjing, China

**Keywords:** HIV prevention, Sub-Saharan Africa, voluntary counselling and testing, determinants, knowledge of HIV, uptake

## Abstract

**Objective:** To examine the major determinants of VCT service uptake among adults in SSA.

**Methods:** Electronic databases were searched to identify eligible English language publications. Reporting of the study selection procedure was done according to PRISMA and the selected articles were also critically appraised.

**Results:** We found 8 significant determinants of VCT uptake among adults in SSA, such as less physical access [OR (Odds ratio): 0.77 (95% CI (Confidence interval): 0.62–0.96), *p* < 0.01], older age [OR: 1.36 (95% CI: 1.08–1.73), *p* < 0.01], higher education level [OR: 1.60 (95% CI: 1.24–2.05), *p* < 0.01], high knowledge of HIV and VCT awareness [OR: 1.40 (95% CI: 1.03–1.90), *p* < 0.01], unprotected sexual practices [OR: 1.75 (95% CI: 1.18–2.58), *p* < 0.01], discussion on HIV among partners and others [OR: 1.76 (95% CI: 1.10–2.81), *p* < 0.01], other STIs [OR: 1.40 (95% CI: 1.00–1.98), *p* < 0.01], and divorced/separated [OR: 1.39 (95% CI: 1.12–1.72), *p* < 0.01].

**Conclusion:** This study showed that 8 determinants were significantly associated with VCT service uptake in SSA. Thus, HIV interventions and policy initiatives should be tailored to these determinants to ensure scale-up of VCT service uptake in SSA.

## Introduction

The global spread of the Human Immunodeficiency Virus (HIV) continues to grow, especially among impoverished populations, despite numerous prevention and control programs implemented over the years. The United Nations Program on AIDS/HIV (UNAIDS) reported an exponential rise in HIV prevalence since the scale-up of antiretroviral therapy (ART) [1]. Approximately 2.2 million new HIV cases have been recorded annually for the past 5 years [2–4], with an estimated 36.8 million people living with HIV (PLHIVs) globally by 2017, and nearly 70% of all the persons living with HIV/AIDS worldwide are in Sub-Saharan Africa (SSA) [1].

Despite the growing awareness of HIV and expanded coverage of HIV Voluntary Counselling and Testing (VCT) services in SSA, about 9.4 million HIV infections in the region remain undiagnosed [1, 5]. Demographic Health Survey (DHS-2013) in The Gambia, for example, showed that 67.4% of all confirmed HIV cases were previously untested [6]. Another study conducted in Ethiopia reported that 33% of health professionals have never tested for HIV [7]. In addition, VCT uptake rates remain low in SSA [4, 8], especially among the youth who are also highly engaged in risky sexual behaviors such as unprotected sexual intercourse, having multiple sexual partners, etc. [9–11]. A study in Nigeria showed that participants aged below 21 years had a decreased odds of having tested for HIV and willing to test for HIV [12, 13]. Moreover, factors such as misconceptions of HIV transmission, fear of testing positive and high stigma, etc. continue to affect VCT uptake rates [1, 11, 13]. Therefore, the UNAIDS 95-95-95 goal by 2030 (95% of all HIV-infected persons should be tested; 95% of those tested should be on ART; and, 95% of those on ART should attain viral suppression) is less likely to be accomplished in SSA.

Low VCT uptake implies that more people living with HIV would not know their status, leading to the onward transmission and increased incidence. It also contributes to late-stage HIV diagnosis and initiation of antiretroviral treatment, which leads to the advancement of the disease to Acquired Immunodeficiency Syndrome (AIDS) and possibly death [14], resulting to increased burden on the health system, as well as a huge economic challenge on the patient/family due to the frequent need for medical care. Moreover, the economic burden may affect the labor market and increase the mortality rate of the country. In addition, the underutilization of VCT services provided at the facility level will lead to a waste of resources due to product expiring before use. This also means that more financial resources need to be expended in developing alternative service provision options to reduce the disease burden in the country. Furthermore, this is a specific dilemma for Low-and-middle Income Countries (LMICs) as the countries already have limited resources available for disease prevention and control [15].

VCT forms a cornerstone of HIV prevention as it offers the opportunity for early diagnosis and treatment [7, 16]. VCT has helped to create awareness of HIV status and provides counselling on risk behavior modification. It has also contributed to decrease stigma and has become a first step to accessing care [17, 18]. However, VCT uptake is dependent on awareness of VCT services availability. Therefore healthcare providers are required to create awareness about service availability to their patients. In addition, they are responsible for instigating provider-initiated testing (they have to offer services to patients who may not know their status). Health care providers bear the responsibility to provide adequate pre and post counseling, and confidentiality of HIV test results. Moreover, due to the high stigma associated with HIV, VCT centers should also be strategically situated and designed to ensure the privacy of testers.

Although previous studies have reported several factors that affect VCT service uptake rate in SSA, such as fear of positive test results, HIV associated social stigma, awareness of VCT services and access to VCT service centers, etc. [19, 20], the evidence remains insufficient and conflicting, thus warranting a systematic review and meta-analysis. The meta-analysis study design provides a more precise estimate of the effect size and may enable the resolution of conflicts between studies yielding to conclusive results [21]. Therefore, our study aims to examine the determinants of VCT service uptake in SSA in a systematic review and meta-analysis. We hope that our findings will contribute to the up-scale of VCT service utilization in SSA by providing viable suggestions on the significant determinants of VCT service uptake to improve HIV intervention programs and policy initiatives.

## Methods

### Search Strategy

We developed a search strategy to identify studies that reported the determinants of Voluntary Counselling and Testing in Sub-Saharan Africa. We searched electronic databases PubMed, Web of Science, Cochrane library, Google Scholar, Science Direct, EMBASE, and African Journals Online for English language literature using the terms “Voluntary Counselling and Testing,” “determinants,” “predictors,” “barriers,” “facilitators,” “community-based VCT,” “provider-initiated VCT,” “adolescents,” “Adults,” and “Sub-Saharan Africa.” The search was conducted between May and June 2019. The search was restricted for articles published from 2010 to 2019. Furthermore, we reviewed reference lists of original and review articles to search for more studies.

### Inclusion and Exclusion Criteria

For inclusion, studies had to fulfill the following criteria: 1) original articles such as cross-sectional studies, case-control, cohort, and experimental studies, 2) assessment of determinants of VCT uptake, including barriers or promoters of VCT uptake, 3) study population is Sub-Saharan Africans, 4) adult or adolescent population. Studies were excluded if: 1) studies were on other HIV testing methods, 2) review articles and letters, 3) articles whose primary population were sex workers, Men having sex with Men (MSM), pregnant women, and 4) lack the relevant effect size estimate [odds ratio (OR)/beta(β), 95%CI, or standard error (SE)]. Sex workers, MSM, and pregnant women were excluded because they are special populations and many studies have been conducted on them.

### Screening and Data Extraction

Screening and data extraction was performed by two independent investigators using Endnote reference manager (Endnote, Version 8), upon exclusion of duplicates, and contradictions between the two investigators were discussed with a third investigator to obtain agreement. The two investigators proceeded to extract relevant data such as the first author’s name and year of publication, study design, participants, sample size, urban/rural residence, the country(s) where the study was conducted, determinants of VCT service uptake, and corresponding effect size estimates, barriers to VCT service uptake, percentage of participants who were ever tested for HIV, percentage of participants with high knowledge of HIV and/or awareness of VCT service, and percentage of participants willing to uptake VCT if offered. The investigators verified each other’s data before finally merging the results. The Preferred Reporting Items for Syatematic Reviews and Meta-analyses (PRISMA) guideline for the reporting of systematic reviews and meta-analyses was adhered to in this study.

### Quality Assessment

Two investigators evaluated the quality of the eligible studies using the Newcastle - Ottawa Quality Assessment Scale (NOS), a validated checklist for assessing the quality of non-randomized studies included in systematic reviews [22]. It consists of several items distributed between three subscales: selection of study groups; the comparability of the groups; and the ascertainment of either the exposure or outcome of interest for case-control or cohort studies respectively. The stars awarded for each quality item serve as a quick visual assessment.

### Meta-Analysis

The predictor variables were summarized into key categories (determinants) with their corresponding effect size estimates (OR (95% CI) in an excel sheet, cross-checked and filtered using excel (Supplementary Table S3). The effect sizes of each study and the corresponding standard error were transformed into their natural logarithms to stabilize the variances and to normalize the distribution. Summary data of the remaining 27 studies were analyzed in R using the “meta-package.” The heterogeneity among studies was quantified by I^2^-statistic and tau^2^ [23]. The Dersimonian-Laird random-effects model was reported where heterogeneity was high (*I* ^*2*^ > 50%, *p* ≤ 0.05) [24].

Furthermore, multivariate meta-regression analysis was performed, using the “metareg” function, to assess possible sources of heterogeneity, such as age, sample size, region, location of the study, place of study, publication date, and population type, at a statistical significance level of *p* ≤ 0.05 (Supplementary File S1). Funnel plot asymmetry was used to detect publication bias, and Egger’s regression test to measure funnel plot asymmetry at a *P*-value < 0.1 [23] (Figure 2). All statistical analyses were performed using Excel and R (R software version 3.6.1) [25].

## Results

**FIGURE 1:**
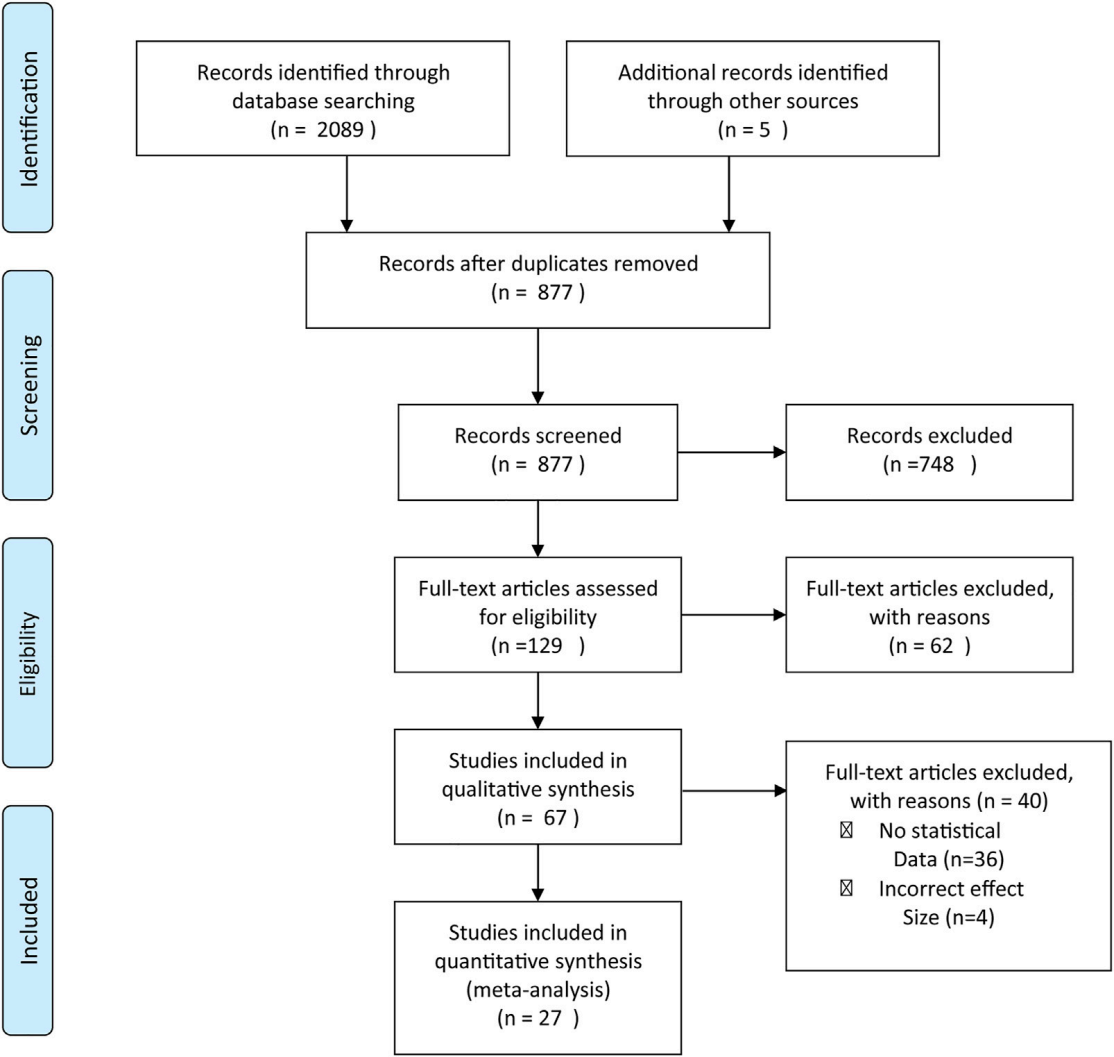
Preferred Reporting Items for Syatematic Reviews and Meta-analyses (PRISMA) flowchart reporting the article selection process (Sub-Saharan Africa. 2020).

Twenty-seven (27) studies fulfilled the inclusion criteria and were included in our meta-analysis. Sixty-two (62) studies were excluded either due to missing information on VCT (other HIV testing methods used), study populations were sex workers, MSM, pregnant women, study populations were non-SSAs, or review articles, etc. Forty (40) articles without the relevant effect size estimates or incorrect effect size estimates were further excluded from the meta-analysis (Figure 1). Eligible articles in this study were published between the years 2012–2019.

Supplementary Table S1 summarizes the characteristics of the included studies. Overall, the meta-analysis included 66,498 participants from 13 Sub-Saharan African countries (6 from Ethiopia, 4 from Uganda and South Africa each, 3 from Mozambique and Nigeria each, and 1 or 2 each from Zimbabwe, Zambia, Tanzania, Congo (Brazzaville), Kenya, Ghana, Cameroon, Burkina Faso, and Malawi. Regionally, 18% of studies (*n* = 5) were from southern Africa followed by East Africa [14% (*n* = 4)], West Africa [11% (*n* = 3)], and Central Africa [7.4% (*n* = 2)]. 55.5% (*n* = 15) of the studies were population/community based while, 44.4% (*n* = 12) were institution based (health facility (*n* = 3), School (*n* = 7), prison, etc.). In terms of study setting, 51.85% (*n* = 14) of the studies were undertaken in urban areas, 22% (*n* = 6) were in rural areas, and 14.81% (*n* = 4) in both rural and urban areas. Cross-sectional studies comprised the majority, 74.10% (*n* = 20), of which 7.4% were national surveys.

Out of 20 studies, the percentage of participants who ever tested for HIV ranged between 2.6% among rural men in Ethiopia [26] and 75% among adults in rural Tanzania [27]. The pooled percentage of participants that uptake VCT was 45.07% (50.15% among young adults), and for studies among young adults alone pooled percentage VCT uptake was 33.30%.

Seven (7) studies reported the percentage of participants with high knowledge of HIV and awareness of VCT services. The pooled “percentage of participants with high knowledge of HIV and awareness of VCT services” and “percentage of participants with willingness for VCT uptake if offered” were 75.79% (ranges, 100%–25.7%; N (Number of studies) = 7) and 63.64%(ranges, 97%–18%; N = 9), respectively. The pooled “percentage of high knowledge of HIV and awareness of VCT services” and “willingness for VCT service uptake” were relatively higher than pooled “percentage of participants who ever uptake VCT service” (45.07%) (Supplementary Table S1).

**FIGURE 2:**
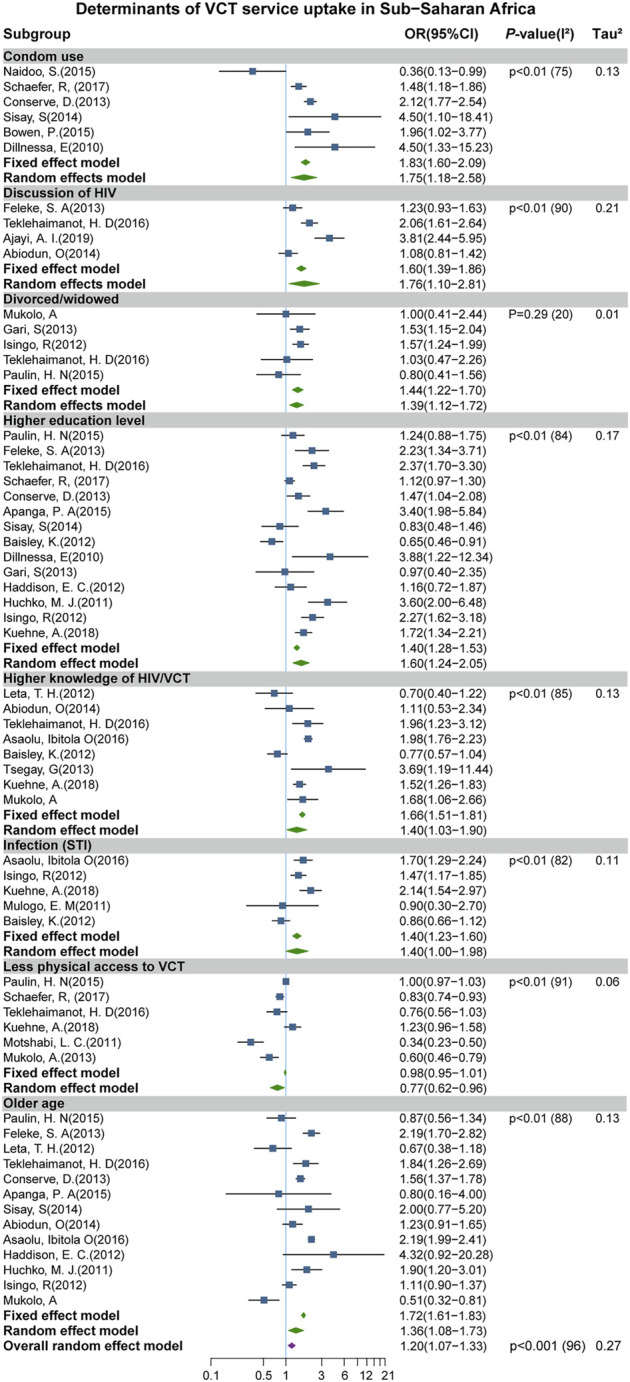
Forest plot showing the significant determinants of Voluntary Counselling and Testing (VCT) service uptake (Sub-Saharan Africa. 2020).

Out of the 14 factors we assessed from the identified studies, our meta-analysis results were significant for the following eight determinants, with their full variable names reported in bracket: condom use (irregular condom use with casual partners) [OR: 1.75 (95%CI: 1.18–2.58)]; discussion of HIV (those that discussed HIV and VCT related issues with partners, friends, family, community, and at school)[OR: 1.76 (95% CI: 1.10–2.81)]; divorced/widowed (respondents’ relationship status) [OR: 1.36 (95% CI: 1.12–1.72)]; higher education level (education level attained) [OR: 1.60 (95% CI: 1.24–2.05)]; higher knowledge of HIV and VCT awareness (knowledge score of respondents) [OR: 1.40 (95% CI: 1.03–1.90)]; infection (STI) (reasons for taking the test) [OR: 1.40 (95%CI: 1–1.98)]; service accessibility (nearness to VCT site) [OR: 0.77 (95% CI: 0.62–0.96)]; and older age (age of the respondent) [OR: 1.36 (95% CI: 1.08–1.73)] (Figure 2).

These findings indicate that individuals who seldom used a condom with casual partners had a 75% increased odds of VCT uptake, compared to those who regularly used a condom. Participants who frequently talk or discuss HIV and VCT related issues (with friends, family, and at schools, etc.) were more likely to uptake VCT. In addition, divorced and widowed women had a higher odds of taking VCT than married women. Adults with higher educational attainment and those with higher knowledge scores on HIV and VCT awareness were more likely to uptake VCT by 60% and 40% than those with lower education levels and less knowledge on HIV and VCT awareness, respectively. Furthermore, we observed that adults who had STI had a 40% increased odds of VCT uptake than those who never had STI. Participants with less access to VCT centers were less likely to uptake VCT services. Also, older aged (>25 years) individuals had a greater odds of VCT service utilization. Therefore, interventions and policy initiatives would realize immense success if tailored to these significant determinants.

However, no significant result was observed among the other six predictors such as “female sex,” “high stigma,” “high wealth index,” “multiple sexual partners,” “perceived At-risk,” and “urban residence.” In addition, no statistically significant result was observed for “religion,” “attitude to VCT,” and “marriage (married vs. single)” subgroups.

**FIGURE 3:**
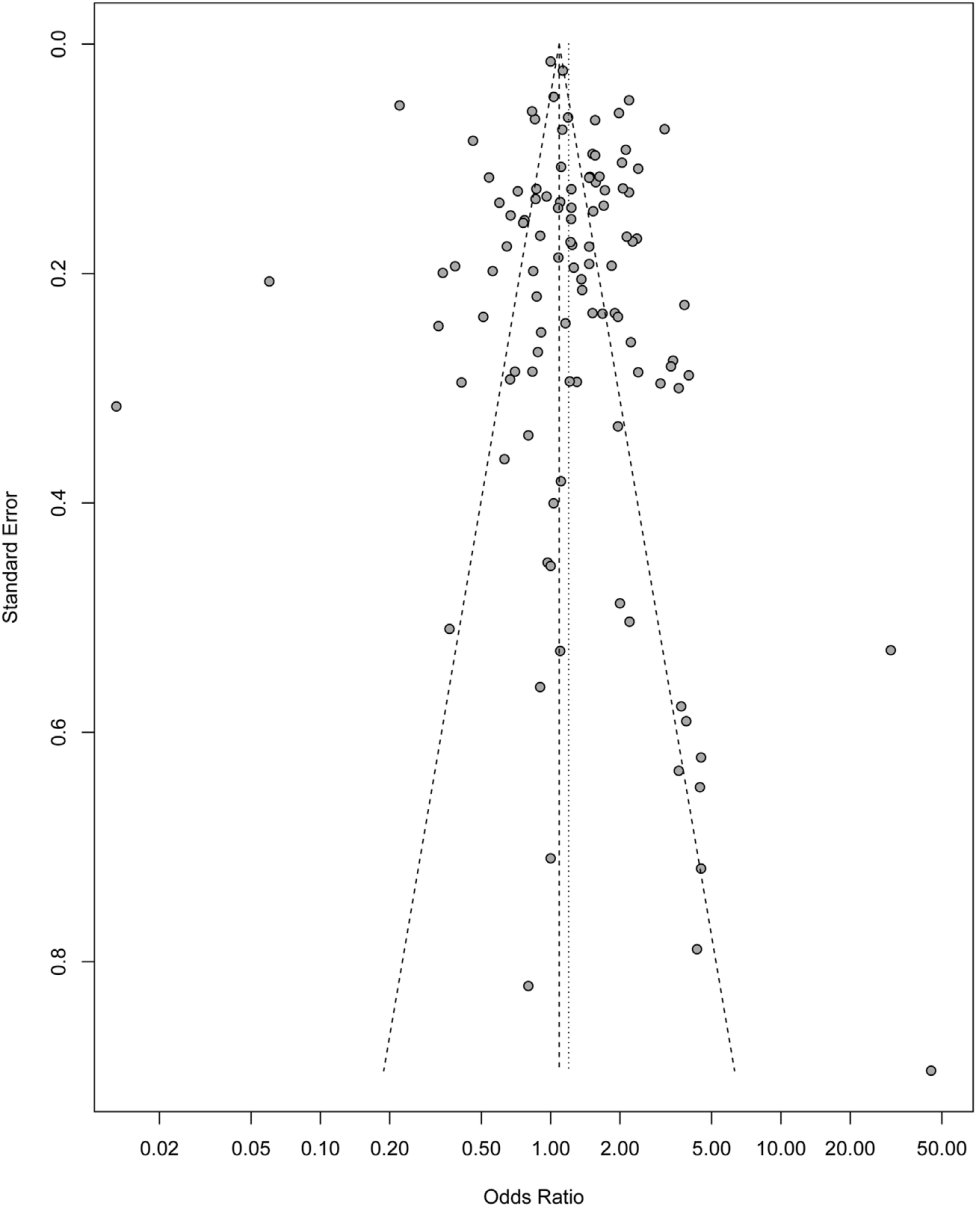
Funnel plot indicating no potential publication bias in the study (Sub-Saharan Africa. 2020).

Study heterogeneity was high (*I* ^2^ > 50%) for most determinants/subgroups (except for divorced/widowed subgroup), suggesting that the studies in this meta-analysis cannot be considered to be from the same population. Thus, we applied a meta-regression analysis to assess the possible sources of heterogeneity among subgroups with more than ten studies, as recommended by the Cochrane guidelines [24]. The meta-regression showed no significant variation by “sample size,” “urban/rural residence,” “female,” “region,” “institution/community based,” and “age (age >30)” for those determinants with high *I* ^2^, except “age (age >30)” in “Older age” subgroup, suggesting that older age >30 years might be the source the observed heterogeneity in the “older age” sub-group (*p* = 0.0097). Although we could not explain the possible sources of the observed heterogeneity in the other subgroups, the linear regression test for asymmetry showed no publication bias in this study (*P*-value = 0.23) (Figure 3).

## Discussion

Our meta-analysis showed significant results for eight [7] determinants of VCT service uptake in SSA (condom use, discussion of HIV, divorced/widowed, higher education level, higher knowledge of HIV and VCT awareness, sexually transmitted infection (STI), less physical access to VCT sites, and older age). Therefore, HIV/AIDS prevention and control interventions programs and policy initiatives ought to be tailored to these factors. A bottom-top approach to these determinants would yield great successes to VCT service utilization in SSA. In addition, government and stakeholder collaboration is also needed in this effort to scale up VCT uptake and reduce HIV transmission.

We observed that adults who attained higher education were approximately twice more likely to uptake VCT services. In agreement with our finding, similar studies reported that secondary school pupils born to educated parents were more likely to accept a VCT service offer [11, 28]. A large percentage of participants willing to utilize VCT service were also reported among students who had participated in educational programs during their primary and junior secondary education levels [29, 30]. We also observed higher odds of VCT uptake among those with increased knowledge of HIV and VCT awareness and routinely discussing HIV issues with partners, family, friends, and at schools. This result is also consistent with findings from a study conducted in Shanghai, China, where rural migrants from sensitized groups were found to be more likely to accept VCT [31]. It is, therefore, evident that strategic HIV sensitization and education can be an impactful facilitator for VCT service uptake if adopted in SSA [13, 32, 33].

Individuals who practice irregular condom use with casual partners had higher odds of VCT uptake. This finding was, however, not surprising as people who engage in risky sexual lifestyles (irregular condom users, multiple sexual partners, sex workers, *etc*.) are usually expected to be more willing to get tested due to the self-perceived risk of high exposure [34–37]. However, we recommend further studies on the impact of lifestyle factors on VCT uptake due to the complexity of this determinant and the high heterogeneity observed in this study.

Furthermore, we observed that divorced/widowed people were more likely to uptake VCT than married women; and, the heterogeneity observed in this subgroup was relatively low (*I* ^2^ = 20%; *p* = 0.29). However, as polygamy and the remarriage of divorced people are common cultural practices in SSA, married people should be encouraged to know their HIV status and maintain regular VCT uptake through the promotion of couple testing. Additionally, the odds of VCT service uptake was higher among older adults (>25 years of age). Similarly a study in Nigeria reported that participants aged <21 years had a decreased odds of having tested for HIV and willing to test for HIV [13]. Although a high heterogeneity was observed in this subgroup, the meta-regression results showed that age >30 years might be the source of the high heterogeneity in this sub-group. Therefore, provision of VCT service centers in secondary schools and universities is recommended to increase access and testing among young adults and adolescents [38]. Abiodun et al., (2014) also suggested innovative school-based HIV/AIDS programs to foster willingness for VCT uptake and periodic HIV testing [13]. People who get infected with other STIs were also more likely to seek VCT services, and our results correlated with findings from a study that showed that most people who test for HIV were motivated by STI or referral by a health professional [39]. Therefore, the inclusion of HIV testing and counseling in routine STI and other screening programs is recommended to increase the number of testers [2] whiles, VCT in the outpatient department and antenatal clinics continue to be promoted.

Our study further showed that adults with less access to VCT service centers (those who live very far away from VCT sites) were less likely to opt for VCT. Several studies also identified the limiting effects of accessibility to VCT services, particularly for rural areas [11, 19, 40, 41]. However, another study in Sierra Leone reported that 90% of participants prefer to test for HIV at VCT service centers that are located very far from their neighborhood to ensure their privacy and confidentiality of test results [42]. This indicates that accessibility alone might not be a standalone determinant; hence, coherent interventions targeted at all the other significant determinants will seem more beneficial. Nonetheless, proper design of VCT centers to ensure privacy is strongly recommended. Health care provides should also be equipped with professional counseling skills, and should offer efficient counseling that would encourage people to opt for VCT services [43]. In addition, community VCT advocacy and anti-HIV stigma campaigns needs to be strengthened and expanded in SSA [7, 26, 44–46]. Furthermore, the formulation of policies and programs to address gender-inequity and confidentiality of HIV test results might enhance VCT service uptake in SSA [4, 43, 47, 48].

We did not observe significant results among the other six factors (“female sex,” “high stigma,” “wealth index,” “multiple sexual partners,” “perceived at risk,” and “urban residence”) although some single studies have reported them as significantly associated with VCT uptake, which could be due to sampling error, study design or time of study. However, future meta-analysis should be directed on these factors.

This meta-analysis provides a synthesis of evidence from 13 different countries across SSA with large sample size. Thus, the evidence from this study can be considered more reliable than those from single studies. In addition, Most of the studies are of good quality, scoring the highest score according to the NOS scale. Although Sub-Saharan African countries share common demographic characteristics, the number of studies from some regions, for example, Southern Africa, was more than others, and some studies had a relatively smaller sample size, and studies with different study designs were included. Also, a high heterogenetic (I^2^) was observed in most subgroups, which could not be accounted for in the meta-regression analysis.

## Conclusion

This study showed that 8 determinants (less physical access, older age, higher education level, high knowledge of HIV and VCT awareness, unprotected sexual practices, discussing HIV topics with partners and others, being divorced/separated, and experience of STIs) were significantly associated with VCT service uptake in SSA. HIV prevention and control interventions and policy initiatives should, therefore, be tailored to these determinants to ensure scale-up of VCT uptake in SSA. More education and awareness of HIV and VCT services are needed, through community-led health education programs, institution and school health, mass media, and the inclusion of HIV awareness in other ongoing health programs. These programs should adopt a bottom-top approach and extend to the remote underserved populations, particularly those with no formal education and having less access to health information, as well as adolescents and young adult populations. For proximity to the population, VCT centers should be established in educational institutions, community centers, market places, youth friendly centers etc. Collaboration with relevant stakeholders in these communities, including Non-Governmental Organizations (NGOs) and civil societies, would also augment HIV programs and upscale VCT uptake. Government policy that provides incentives for HIV testing would promote VCT uptake in SSA.

## Data Availability

The dataset(s) supporting the conclusion of this article is included within the article/Supplementary Material.
