# Supplementary material for: Determinants of Voluntary Counseling and Testing Service Uptake Among Adult Sub-Saharan Africans: A Systematic Review and Meta-Analysis
Source: Public Health Rev. 2022 Aug 3;43:1604065. doi: 10.3389/phrs.2022.1604065 (PMC9381697; doi:10.3389/phrs.2022.1604065)
Supplement: Supplementary file 1 [file DataSheet1.doc]

**Supplementary File**

**Determinants of Voluntary Counseling and Testing (VCT) Service Uptake Among Adult Sub-Saharan Africans: A Systematic Review and Meta-analysis.**

**Captions**

Table S1. Description of studies included in the meta-analysis.

Table S2. Critical appraisal checklist for selected articles according Newcastle Critical Appraisal guidelines.

Table S3. Table for preparation of data into summary categorical predictors.

Table S4: Summary of categorical predictors.

File S1. Meta-regression results

Table S5. Results of the 14 categorical determinants of VCT service Uptake in SSA identified from literature.

Figure S1: Forest plot showing results of the 14 categorical determinants of VCT service Uptake in SSA identified from literature.

Table S6. PRISMA checklist for reporting of systematic reviews and meta-analysis

| **Table S1. Description of studies included in the meta-analysis** | | | | | | | |  | | | |
| --- | --- | --- | --- | --- | --- | --- | --- | --- | --- | --- | --- |
| Author (year) | Participants | Design | Sample size | Urban/Rural | Country | Determinants of VCT service uptake | Effect size estimate (β/ OR (95%CI) | Barriers to VCT service uptake | VCT uptake (%) | Awareness of HIV /VCT services (%) | Willingness for VCT uptake (%) |
| Abamecha, F (2013) | Health Professionals | Health facility Based | 336 | Urban | Ethiopia | Perceived at risk of HIV | 0.55 (−0.06, - 0.12); β | Negative attitude and perceived social pressure | 33 | 100 | 30.3 |
| Abiodun, O (2014) | University Students | University | 1250 | Urban | Nigeria | Higher knowledge of HIV/AIDS | 1.11 (0.53, 2.34) | Awareness of VCT service; male gender is less likely to uptake the VCT | 30.4 | 95 | 72 |
|  |  |  |  |  |  | Age groups (<21) | 0.82 (0.60, 1.10) |  |  |  |  |
|  |  |  |  |  |  | Sex (Female) | 0.87 (0.68, 1.11) |  |  |  |  |
|  |  |  |  |  |  | Christianity | 0.74 (0.46, 1.20) |  |  |  |  |
| Ajayi, A. I.(2019) | University students (Young Adults) | University | 833 | Urban | South African | Highly concerned about contracting HIV/STIs | 4.28 (2.50, 7.34) | Fear of testing positive; Never tested for HIV before | 58.4 | / | / |
|  |  |  |  |  |  | Knowing one's partner's status | 3.81 (2.44, 5.96) |  |  |  |  |
|  |  |  |  |  |  | Discussion of HIV/STIs with partner | 1.83 (1.20, 2.80) |  |  |  |  |
|  |  |  |  |  |  | Having received VCT in the past | 2.66 (1.77, 3.99) |  |  |  |  |
| Apanga, P. A (2015) | Adults (18-55) | Community based | 200 | Urban and Rural | Ghana | Age (21-37) | 0.8 (0.16, 2.52) | Lack of awareness of the VCT service in the area; fear of being stigmatized; belief that HIV/AIDS cannot be treated | 70 | 90 | 97 |
|  |  |  |  |  |  | Female | 1.1 (0.39, 2.90) |  |  |  |  |
|  |  |  |  |  |  | Married (ref. unmarried) | 1.1 (0.41, 2.69) |  |  |  |  |
|  |  |  |  |  |  | Religion (Christianity) | 0.9 (0.18, 2.38) |  |  |  |  |
|  |  |  |  |  |  | Education level (Tertiary) | 3.4(1.98, 8.42) |  |  |  |  |
| Asaolu, Ibitola O (2016). | Youth aged (15–24 years) | Community based | 23, 367 | National (DHS) | Nigeria, Mozambique, Congo(Brazzaville) and Uganda | Older youth | 2.19 (1.99, 2.40) | Poor knowledge of HIV/AIDS | 36.5 | 25.7 | 50.3 |
|  |  |  |  |  |  | Comprehensive knowledge of HIV | 1.98 (1.76, 2.22 ) |  |  |  |  |
|  |  |  |  |  |  | More sexual partners | 0.46 (0.39, 0.54) |  |  |  |  |
|  |  |  |  |  |  | Infection (STI) | 1.70 (1.29, 2.24) |  |  |  |  |
|  |  |  |  |  |  | Sex (Male) | 0.32 ( 0.28, 0.37) |  |  |  |  |
| Baisley, K. (2012) | Youth (22-23) | Community based | 12590 | Rural | Tanzania | Infection (STI) | 0.86 (0.66, 1.14) | For females (decreased knowledge of HIV acquisition, sex with a casual partner, and being HSV-2 seronegative); for males (lower education and having more lifetime sexual partners). | 75 | / | 90 |
|  |  |  |  |  |  | Higher education | 0.65 (0.45, 0.90) |  |  |  |  |
|  |  |  |  |  |  | More sexual partners (>3) | 0.84 (0.57, 1.23) |  |  |  |  |
|  |  |  |  |  |  | Religion (Islam) | 0.77 (0.57, 1.16) |  |  |  |  |
|  |  |  |  |  |  | Perceived at risk | 1.52 (0.96, 2.38) |  |  |  |  |
|  |  |  |  |  |  | Higher knowledge (>3 correct answers) | 0.77 (0.57, 1.05) |  |  |  |  |
|  |  |  |  |  |  | Married (ref. unmarried) | 1.04 (0.90, 1.21) |  |  |  |  |
| Bowen, P.(2015) | Construction workers | Community based | 512 | Urban | South Africa | Sex (Females) | 4.45 (1.25, 15.82) | Male gender, temporal employment, irregular condom use | 73 | / | / |
|  |  |  |  |  |  | Regular condom use | 1.93 (1.02-3.65) |  |  |  |  |
| Conserve, D.(2013). | Adult men(>15years) | Community based. | 9107 | Urban | Uganda | Multiple sexual partners | 0.54 (0.43, 0.67) | Multiple sexual partners | 35.5 | / | / |
|  |  |  |  |  |  | Regular condom use | 2.13 (1.77, 2.55) |  |  |  |  |
|  |  |  |  |  |  | Perceived at risk of HIV | 0.22 (0.20, 0.25) |  |  |  |  |
|  |  |  |  |  |  | Married (ref. unmarried) | 0.55 (0.47, 0.65) |  |  |  |  |
|  |  |  |  |  |  | Higher education level | 1.48 (1.04, 2.09) |  |  |  |  |
|  |  |  |  |  |  | Older age | 1.56 (1.37, 1.81) |  |  |  |  |
| Daniyam, C. A. (2010) | Clinical Medical Students | University | 368 | Urban | Nigeria | Sex (female) | 0.63 (0.31, 1.260 | Fear of a positive test result | 50.7 | High | 83.1 |
| Dillnessa, E (2010) | Couples | Health facility based | 771 | Urban | Ethiopia | Higher education level | 3.88 (1.22, 12.3) | knowledge/awareness of VCT service; live outside home for a long time | 14.5 | / | / |
|  |  |  |  |  |  | Regular condom use | 4.59 (1.33, 15.31) |  |  |  |  |
| Feleke, S. A (2013) | Adolescents (15-19 years old) | Community based | 1290 | Urban | Ethiopia | Perceived at risk of HIV | 30 (10.65, 83.01) | Access to VCT service; perceived risk towards HIV/AIDs. | 72.2 | High | / |
|  |  |  |  |  |  | Discussion of HIV/VCT | 1.23 (0.93,1.69) |  |  |  |  |
|  |  |  |  |  |  | Sex (female) | 0.38 (0.26,0.56) |  |  |  |  |
|  |  |  |  |  |  | Higher education level | 2.23 (1.34, 3.72) |  |  |  |  |
|  |  |  |  |  |  | Older age | 2.19 (1.70, 2.83) |  |  |  |  |
| Gari, S (2013) | Adults | Community-based | 1716 | Urban and Rural | Zambia | Higher education Level | 0.97 (0.4, 0.97) | Fear of social rejection; disruptive couple relationships; tolerance to gender-based violence (men); fear to take medication; unawareness of ART; and Poverty. | 69 | / | / |
|  |  |  |  |  |  | Married (ref. unmarried) | 2.5 (2.04, 3.125) |  |  |  |  |
|  |  |  |  |  |  | Divorced/ Widowed | 1.53 (1.15, 2.05) |  |  |  |  |
|  |  |  |  |  |  | Sex (Female) | 0.67 (0.5, 0.75) |  |  |  |  |
|  |  |  |  |  |  | Wealth index | 0.85 (0.75, 0.98) |  |  |  |  |
|  |  |  |  |  |  | Urban residence | 2.4 (1.94, 2.96) |  |  |  |  |
| Haddison, E. C.(2012) | High school students | School | 474 | Rural | Cameroon | Older age (23-25 years) | 1.4.3 (0.92, 20.3) | Do not know where to go for the VCT | 27.8 | 73.8 | 69.4 |
|  |  |  |  |  |  | Higher education level | 1.16 (0.72, 1.88) |  |  |  |  |
|  |  |  |  |  |  | Positive attitude | 2.07 (0.99, 4.34) |  |  |  |  |
|  |  |  |  |  |  | Perceived at risk | 1.21 (0.68, 2.14) |  |  |  |  |
|  |  |  |  |  |  | Sex (female) | 3.33 (1.92, 5.0) |  |  |  |  |
| Huchko, M. J.(2011) | Adults(15–49) | Community-based | 1655 | Urban | Kenya | Older age | 1.90 (1.20, 2.90) | Access to VCT service; fear of disclosure of HIV status; HIV testing in Antenatal Care Centers | 59 | / | / |
|  |  |  |  |  |  | Higher education level | 3.60 (2.00, 6.60) |  |  |  |  |
|  |  |  |  |  |  | Married | 0.70 (0.50, 0.90) |  |  |  |  |
|  |  |  |  |  |  | Religion (Islam) | 0.77 (0.40, 1.66) |  |  |  |  |
| Isingo, R (2012) | Adults | Health center | 9457 | Rural | Tanzania | Infection (STI) | 1.47 (1.17, 1.84) | HIV-related stigma; and concerns over confidentiality at VCT service centers. | 26 | / | 18 |
|  |  |  |  |  |  | More sexual partners(>3) | 3.98 (2.26, 7.01) |  |  |  |  |
|  |  |  |  |  |  | Urban residence | 1.09 (0.72, 1.65) |  |  |  |  |
|  |  |  |  |  |  | Divorced/Widowed | 1.13 (1.08, 1.58) |  |  |  |  |
|  |  |  |  |  |  | Married (ref. unmarried) | 1.57 (1.24, 20) |  |  |  |  |
|  |  |  |  |  |  | Higher education level | 0.99 (0.75, 1.31) |  |  |  |  |
|  |  |  |  |  |  | Older age | 2.27 (1.62, 3.18) |  |  |  |  |
| Kuehne, A.(2018) | Migrant Sub-Saharan Africa | Community-based | 2782 | Urban | Germany | Infection (STI) | 2.14 (1.54, 2.97) | Inconsistent condom use, and multiple sexual partners; lower knowledge of HIV/AIDs. | 60.9 | 77.2 (female) & 74 (Male) | / |
|  |  |  |  |  |  | Perceived at risk | 0.72 (0.56-0.92) |  |  |  |  |
|  |  |  |  |  |  | Religion (Islam) | 0.66 (0.53, 0.81) |  |  |  |  |
|  |  |  |  |  |  | More sexual partners | 1.23 (0.96, 1.60) |  |  |  |  |
|  |  |  |  |  |  | Higher wealth index | 1.10 (0.84, 1.48) |  |  |  |  |
|  |  |  |  |  |  | Sex (women) | 1.56 (1.29, 1.88) |  |  |  |  |
|  |  |  |  |  |  | Higher stigma | 1.60 (1.30, 2.03) |  |  |  |  |
|  |  |  |  |  |  | Higher knowledge | 1.52 (1.26, 1.84) |  |  |  |  |
|  |  |  |  |  |  | Higher education level | 1.72 (1.34, 2.19) |  |  |  |  |
| Tsegay, G(2013) | University Students, | University | 711 | Urban | Ethiopia | Knowledgeable of HIV/AIDs | 3.69 (1.19,11.49) | Perceived stigma; not feeling at risk and trusting one-self and their sexual partner; unavailability of VCT service in the area; no knowledge of where to take the VCT; busyness; sexual partner influence. | 58.5 | 85.4 | / |
|  |  |  |  |  |  | High stigma | 0.01 (0.01, 0.02) |  |  |  |  |
|  |  |  |  |  |  | Perceived at risk | 2.40 (1.37, 4.23) |  |  |  |  |
|  |  |  |  |  |  | Attitude | 3.00 (1.65, 5.49) |  |  |  |  |
| Teklehaimanot, H. D(2016) | Adults | Community based | 11919 | Rural | Ethiopia | Older age | 1.84 (1.26-2.68 | Stigma; Poverty; lack of communication between partners about HIV; low knowledge of HIV and about VCT service. | 25.8 | / | / |
|  |  |  |  |  |  | Higher wealth index | 1.39 (0.73-2.29) |  |  |  |  |
|  |  |  |  |  |  | Discussion of HIV/VCT | 2.06 (1.61-2.71) |  |  |  |  |
|  |  |  |  |  |  | Perceived at risk | 1.26 (0.86-1.84) |  |  |  |  |
|  |  |  |  |  |  | Multiple sexual partners | 0.76 (0.56-1.03) |  |  |  |  |
|  |  |  |  |  |  | Sex (Female) | 0.90 (0.6-1.25) |  |  |  |  |
|  |  |  |  |  |  | High stigma | 0.56 (0.38-0.8) |  |  |  |  |
|  |  |  |  |  |  | Divorced/ Widowed | 1.03 (0.47-2.26) |  |  |  |  |
|  |  |  |  |  |  | Marriage | 0.76 (0.54-1.09) |  |  |  |  |
|  |  |  |  |  |  | Higher Knowledge | 1.96 (1.23-3.13) |  |  |  |  |
|  |  |  |  |  |  | Higher education level | 2.37 (1.7-3.140 |  |  |  |  |
| Leta, T. H.(2012). | Men(15-59) | Community based | 6778 | Urban and rural | Ethiopia | Older age | 0.66 (0.30, 1.23) | HIV/AIDS-related knowledge index; lower Socio-economic position; ever married. | 21.9 (urban) & 2.6 ( rural men) | / | / |
|  |  |  |  |  |  | Higher knowledge of HIV | 0.77 (0.4, 1.33) |  |  |  |  |
|  |  |  |  |  |  | Marriage | 1.43 (0.78, 2.94) |  |  |  |  |
|  |  |  |  |  |  | High stigma | 3.60 (1.04, 2.46) |  |  |  |  |
|  |  |  |  |  |  | Higher wealth index | 2.20 (0.82, 5.94) |  |  |  |  |
|  |  |  |  |  |  | Perceived at risk | 3.0 (1.68, 5.51) |  |  |  |  |
| Motshabi, L. C. (2011). | Male adult inmates | Prison | 200 | Urban | South Africa | More physical access | 0.34 ( 0.23, 0.50) | Poor health system | / | / | / |
| Schaefer, R, (2017). | Adults | community based | 8092 | Rural &urban | Zimbabwe | Multiple sexual partners | 1.22 (0.87, 1.71) | Recent migrants; younger age and marriage | / | / | / |
|  |  |  |  |  |  | Regular condom use | 1.48 (1.18, 1.86) |  |  |  |  |
|  |  |  |  |  |  | More physical access | 0.83 (0.74, 0.94) |  |  |  |  |
|  |  |  |  |  |  | Urban residence | 0.06 (0.04, 0.08) |  |  |  |  |
|  |  |  |  |  |  | High wealth index | 2.40 (1.66, 2.5) |  |  |  |  |
|  |  |  |  |  |  | Sex (Female) | 1.19 (1.05, 1.35) |  |  |  |  |
|  |  |  |  |  |  | Higher education level | 1.12 (0.97, 1.32) |  |  |  |  |
| Sisay, S (2014). | Secondary school students | High school | 339 | Urban | Ethiopia | Older age | 1. 20 (0.77, 3.33) | Never had sex; fear of HIV; and fear of stigma | / | / | / |
|  |  |  |  |  |  | Higher education level | 0.83 (0.47, 1.42) |  |  |  |  |
|  |  |  |  |  |  | Sex (Female) | 0.97 (0.55, 1.43) |  |  |  |  |
|  |  |  |  |  |  | Perceived at risk | 1.0 (0.25, 3.33) |  |  |  |  |
|  |  |  |  |  |  | Regular condom use | 4.5 (1.1, 23.3) |  |  |  |  |
| Mulogo, E. M (2011) | Adults | Health facility and community based | 994 | Rural | Uganda | Infection (STI) | 0.90 (0.30- 2.70) | Stigma (Family will not treat you as a full family member, Friends will banish you, Community will treat you as an outcast, and family will not care for you) | / | / | / |
| Mukolo, A.(2013) | Female heads of households | Community based | 3749 | Rural and urban | Mozambique | Higher knowledge Older age | 0.68 (1.06, 2.68) 0.51(0.31, 0.81) | Perceived stigma; low knowledge of HIV/AIDS. | 13 | / | 63 |
|  |  |  |  |  |  | Divorced/Widowed | 3. 10 (0.41, 2.43) |  |  |  |  |
|  |  |  |  |  |  | High stigma | 0.88 (0.52, 1.47) |  |  |  |  |
|  |  |  |  |  |  | Urban residence | 1.47 (1.01, 2.13) |  |  |  |  |
|  |  |  |  |  |  | More physical access | 0.59 (0.45, 7.87) |  |  |  |  |
|  |  |  |  |  |  | Religion (Islam) | 0.90 (0.59, 1.35) |  |  |  |  |
|  |  |  |  |  |  | Positive attitude for VCT | 7.61 (5.08, 11.4) |  |  |  |  |
| Obermeyer, C. M (2013) | Adults | Community based | 3659 | National | Burkina Faso, Kenya, Malawi and Uganda | Perceived at risk | 1.03 (0.94, 1.13) | No formal education and awareness level | / | / | / |
| Paulin, H. N (2015) | Female heads of household | Community based | 3708 | Rural | Mozambique | Older age | 0.86 (0.56, 1.33) | Higher HIV-associated stigma | / | / | / |
|  |  |  |  |  |  | Higher education level | 1.24 (0.88, 1.75) |  |  |  |  |
|  |  |  |  |  |  | Married | 1.35 (0.98, 1.86) |  |  |  |  |
|  |  |  |  |  |  | Divorced/Widowed | 0.80 (0.41, 1.86) |  |  |  |  |
|  |  |  |  |  |  | High stigma | 0.41 (0.23, 0.71) |  |  |  |  |
|  |  |  |  |  |  | Sex (Female) | 1.08 (0.75, 1.56) |  |  |  |  |
|  |  |  |  |  |  | High wealth index | 1.37 (0.9, 2.08) |  |  |  |  |
|  |  |  |  |  |  | Urban residence | 1.36 (0.91, 2.04) |  |  |  |  |
|  |  |  |  |  |  | More physical access | 1.0 (0.97, 1.03) |  |  |  |  |
|  |  |  |  |  |  | Religion(Islam) | 1.02 (0.59, 1.76) |  |  |  |  |
| Naidoo, S.(2015) | High school students | School based | 1114 (high school ) | Urban | South Africa | Sex (Female) | 44.9 (7.77, 259.38) | Practicing unsafe sex; male were less like to have been tested | / | / | / |
|  |  |  |  |  |  | Regular condom use | 0.36 (0.13, 0.99) |  |  |  |  |
|  |  |  |  |  |  | Positive attitude for VCT | 15.28 (5.16, 45.23) |  |  |  |  |
| N=Number of participants; /=indicate unavailable data; ART=Antiretroviral Therapy; VCT=Voluntary Counselling and Testing; HIV=Human Immunodeficiency Virus; TB= Tuberculosis; MisSA=Migrants from Sub-Saharan Africa; β=beta; 95%CI=95% confidence Interval. Willingness for VCT uptake (%) = Percentage number of participants in a study willing to uptake VCT if offered; Awareness of HIV /VCT services (%) =percentage number of participants in a study having high knowledge or awareness of HIV and/VCT services; VCT uptake (%) = Percentage number of participants in a study who ever tested for HIV; Country=country of origin where study was undertaken. | | | | | | | | | | | |

| **Table S2. Critical appraisal checklist for selected articles according to** Newcastle - Ottawa Quality Assessment Scale | | | | |
| --- | --- | --- | --- | --- |
| **NO.** | **FIRST AUTHOR (YEAR)** | **Selection (5 * max)** | **Comparability (2 * max)** | **Outcome (3* max)** |
| Cross-sectional studies | | | | |
| 1 | Abamecha, F(2013) | **** | ** | ** |
| 2 | Abiodun, O (2014) | **** | ** | ** |
| 3 | Ajayi, A. I. (2019) | ***** | ** | *** |
| 4 | Apanga, P. A (2015) | **** | ** | ** |
| 5 | Asaolu, Ibitola O (2016) | **** | ** | *** |
| 6 | Bowen, P. (2015) | ***** | ** | *** |
| 7 | Daniyam, C. A. (2010) | *** | ** | *** |
| 8 | Dillnessa, E (2010) | ***** | ** | *** |
| 9 | Feleke, S. A (2013) | ***** | ** | *** |
| 10 | Gari, S (2013) | ***** | ** | *** |
| 11 | Haddison, E. C. (2012) | ***** | ** | *** |
| 12 | Huchko, M. J. (2011) | ***** | ** | *** |
| 13 | Isingo, R (2012) | ***** | ** | *** |
| 14 | Kuehne, A. (2018) | ***** | ** | *** |
| 15 | Tsegay, G (2013) | ***** | ** | *** |
| 16 | Teklehaimanot, H. D (2016) | ***** | ** | *** |
| 17 | Leta, T. H. (2012) | ***** | ** | *** |
| 18 | Sisay, S (2014) | ***** | ** | *** |
| 19 | Mukolo, A. (2013) | **** | ** | *** |
| 20 | Obermeyer, C. M (2013) | ***** | ** | *** |
| 21 | Paulin, H. N (2015) | ***** | ** | *** |
| 22 | Naidoo, S. (2015) | ***** | ** | *** |
| 23 | Conserve, D. (2013) | **** | ** | *** |
| Community-trial | | | | |
| 24 | Baisley, K. (2012) | ***** | ** | *** |
| Case- control  **exposure** | | | | |
| 25 | Motshabi, L. C. (2011) | **** | ** | *** |
| Cohort studies  **Outcome** | | | | |
| 26 | Schaefer, R, (2017) | ***** | ** | *** |
| 27 | Mulogo, E. M (2011) | ***** | ** | *** |
| * =The stars awarded for each quality item serve as a quick visual assessment | | | | |

**Table S3. Table for preparation of data into summary categorical determinants**

| # | Ref. Predictors | Ref. no | Author | Determinants from the Studies | Determinants category | Sample size | OR | Confidence interval | | Beta (β) | Standard error (SE) | P-value | Quality control(UL) |
| --- | --- | --- | --- | --- | --- | --- | --- | --- | --- | --- | --- | --- | --- |
|  |  |  |  |  |  |  |  | Lower limit | Upper limit |  |  |  |  |
| 1 | 20 years (ref) | 1 | Paulin, H. N(2015) | AGE(50 years) | Older age | 3708 | 0.86 | 0.56 | 1.33 | -0.14 | 0.22 | 0.29 | 1.34 |
|  | 15-16 | 2 | Feleke, S. A(2013) | 17-19 | Older age | 1290 | 2.19 | 1.7 | 2.81 | 0.78 | 0.13 | 1.04 | 2.82 |
|  | 25–34 years | 9 | Leta, T. H.(2012) | 45-59 years | Older age | 6778 | 0.66 | 0.37 | 1.23 | -0.40 | 0.29 | 0.17 | 1.18 |
|  | 15-19 | 10 | Teklehaimanot, H. D(2016) | 25-29 | Older age | 11919 | 1.84 | 1.26 | 2.68 | 0.61 | 0.19 | 0.99 | 2.69 |
|  | <26 years | 26 | Conserve, D.(2013) | >26 years | Older age | 9107 | 1.56 | 1.37 | 1.81 | 0.44 | 0.07 | 0.57 | 1.77 |
|  | 18-27 | 24 | Apanga, P. A(2015) | 28-37 | Older age | 200 | 0.8 | 0.16 | 2.52 | -0.22 | 0.82 | 1.38 | 4.0 |
|  | 15-19 | 23 | Sisay, S(2014) | 20-24 | Older age | 339 | 2 | 0.77 | 3.33 | 0.69 | 0.49 | 1.65 | 5.20 |
|  | Less than 21 years | 22 | Abiodun, O(2014) | more than 21 | Older age | 1250 | 1.23 | 0.91 | 1.66 | 0.20 | 0.15 | 0.50 | 1.65 |
|  | <15years | 20 | Asaolu, Ibitola O(2016) | older youth (>19 and above) | Older age | 23367 | 2.19 | 1.99 | 2.40 | 0.78 | 0.05 | 0.88 | 2.41 |
|  | 14-16 | 15 | Haddison, E. C.(2012) | 23-25 | Older age | 474 | 4.32 | 0.92 | 20.3 | 1.46 | 0.79 | 3.01 | 20.29 |
|  | 15-19 | 14 | Huchko, M. J.(2011) | 25-29 | Older age | 1655 | 1.9 | 1.2 | 2.9 | 0.64 | 0.23 | 1.10 | 3.01 |
|  | 15-24 | 13 | Isingo, R(2012) | 25-34 | Older age | 9457 | 1.11 | 0.9 | 1.36 | 0.10 | 0.11 | 0.31 | 1.37 |
|  | <age | 28 | Mukolo, A | per 10 years increase | Older age | 2749 | 0.51 | 0.32 | 0.81 | -0.67 | 0.24 | -0.21 | 0.81 |
|  |  |  |  |  |  |  |  |  |  |  |  |  |  |
| 2 | No education(0 ref) | 1 | Paulin, H. N(2015) | Five years in school | Higher education level | 3708 | 1.24 | 0.88 | 1.75 | 0.22 | 0.17 | 0.56 | 1.75 |
|  | No formal education | 2 | Feleke, S. A(2013) | Primary education | Higher education level | 1290 | 2.23 | 1.34 | 3.72 | 0.80 | 0.26 | 1.31 | 3.71 |
|  | None | 10 | Teklehaimanot, H. D(2016) | Secondary and higher | Higher education level | 11919 | 2.37 | 1.7 | 3.14 | 0.86 | 0.17 | 1.19 | 3.30 |
|  | Primary education | 4 | Schaefer, R, (2017) | Secondary education | Higher education level | 8092 | 1.12 | 0.97 | 1.32 | 0.12 | 0.07 | 0.26 | 1.30 |
|  | No formal education | 26 | Conserve, D.(2013) | Secondary & higher | Higher education level | 107 | 1.47 | 1.04 | 2.09 | 0.39 | 0.18 | 0.73 | 2.08 |
|  | No formal education | 24 | Apanga, P. A(2015) | Tertiary | Higher education level | 200 | 3.4 | 1.98 | 8.42 | 1.22 | 0.28 | 1.76 | 5.84 |
|  | Grade(9-10) | 23 | Sisay, S(2014) | Grade(11 - 12) | Higher education level | 339 | 0.83 | 0.48 | 1.43 | -0.18 | 0.29 | 0.38 | 1.46 |
|  | Primary education | 19 | Baisley, K.(2012) | Secondary education | Higher education level | 12590 | 0.65 | 0.46 | 0.91 | -0.44 | 0.18 | -0.09 | 0.91 |
|  | No education | 17 | Dillnessa, E(2010) | Primary education | Higher education level | 771 | 3.88 | 1.22 | 12.3 | 1.36 | 0.59 | 2.51 | 12.34 |
|  | None | 16 | Gari, S(2013) | Secondary | Higher education level | 1716 | 0.97 | 0.4 | 0.97 | -0.03 | 0.45 | 0.86 | 2.35 |
|  | Lower six grade | 15 | Haddison, E. C.(2012) | Upper six grade | Higher education level | 474 | 1.16 | 0.72 | 1.88 | 0.15 | 0.24 | 0.63 | 1.87 |
|  | No education | 14 | Huchko, M. J.(2011) | College/university | Higher education level | 1655 | 3.6 | 2 | 6.6 | 1.28 | 0.29 | 1.87 | 6.48 |
|  | No formal education | 13 | Isingo, R(2012) | Secondary | Higher education level | 9457 | 2.27 | 1.62 | 3.18 | 0.82 | 0.17 | 1.16 | 3.18 |
|  | No formal education | 12 | Kuehne, A.(2018) | University/college | Higher education level | 9457 | 1.72 | 1.34 | 2.19 | 0.54 | 0.13 | 0.79 | 2.21 |
|  |  |  |  |  |  |  |  |  |  |  |  |  |  |
|  | Low knowledge | 9 | Leta, T. H.(2012) | Comprehensive knowledge | Higher knowledge of HIV/VCT | 6778 | 0.7 | 0.4 | 1.33 | -0.36 | 0.29 | 0.20 | 1.23 |
|  | Low knowledge | 22 | Abiodun, O(2014) | Higher | Higher knowledge of HIV/VCT | 1250 | 1.108 | 0.525 | 2.34 | 0.10 | 0.38 | 0.85 | 2.34 |
|  | None | 10 | Teklehaimanot, H. D(2016) | Comprehensive knowledge | Higher knowledge of HIV/VCT | 11919 | 1.96 | 1.23 | 3.13 | 0.67 | 0.24 | 1.14 | 3.12 |
|  | No | 20 | Asaolu, Ibitola O(2016) | Comprehensive knowledge | Higher knowledge of HIV/VCT | 23367 | 1.98 | 1.76 | 2.22 | 0.68 | 0.06 | 0.80 | 2.23 |
|  | 0-2 correct answers | 19 | Baisley, K.(2012) | 3 correct answers | Higher knowledge of HIV/VCT | 12590 | 0.77 | 0.57 | 1.05 | -0.26 | 0.15 | 0.04 | 1.04 |
|  | Not knowledge of HIV | 11 | Tsegay, G(2013) | Knowledgeable | Higher knowledge of HIV/VCT | 711 | 3.69 | 1.19 | 11.49 | 1.31 | 0.58 | 2.44 | 11.4 |
|  | No | 12 | Kuehne, A.(2018) | Knowledge of VCT(yes) | Higher knowledge of HIV/VCT | 9457 | 1.52 | 1.26 | 1.84 | 0.42 | 0.09 | 0.61 | 1.83 |
|  | 1pt | 28 | Mukolo, A | HIV knowledge score (5 versus 1 pt) | Higher knowledge of HIV/VCT | 2749 | 1.68 | 1.06 | 2.68 | 0.52 | 0.23 | 0.98 | 2.66 |
|  |  |  |  |  |  |  |  |  |  |  |  |  |  |
| 4 | Married (ref.) | 1 | Paulin, H. N(2015) | Single | Being married | 3708 | 1.35 | 0.98 | 1.86 | 0.30 | 0.16 | 0.62 | 1.86 |
|  | Married | 9 | Leta, T. H.(2012) | Never married | Being married | 6778 | 1.43 | 0.78 | 2.94 | 0.36 | 0.31 | 0.96 | 2.6 |
|  | Married | 10 | Teklehaimanot, H. D(2016) | Never married | Being married | 11919 | 0.76 | 0.54 | 1.09 | -0.27 | 0.17 | 0.07 | 1.07 |
|  | Married | 26 | Conserve, D.(2013) | Single | Being married | 9107 | 0.55 | 0.48 | 0.65 | -0.59 | 0.08 | -0.44 | 0.64 |
|  | Married | 24 | Apanga, P. A(2015) | Single | Being married | 200 | 0.91 | 0.37 | 2.44 | -0.09 | 0.46 | 0.79 | 2.22 |
|  | Married | 19 | Baisley, K.(2012) | Single | Being married | 12590 | 1.04 | 0.91 | 1.22 | 0.04 | 0.07 | 0.19 | 1.20 |
|  | Married | 16 | Gari, S(2013) | Single | Being married | 1716 | 2.5 | 2.04 | 3.135 | 0.92 | 0.10 | 1.12 | 3.06 |
|  | Ever married | 14 | Huchko, M. J.(2011) | Single | Being married | 1655 | 0.7 | 0.5 | 0.9 | -0.36 | 0.17 | -0.02 | 0.98 |
|  | Married monogamously | 13 | Isingo, R(2012) | Single never married | Being married | 9457 | 0.99 | 0.75 | 1.31 | -0.01 | 0.14 | 0.27 | 1.31 |
|  |  |  |  |  |  |  |  |  |  |  |  |  |  |
| 5 | Married | 28 | Mukolo, A | Divorced/widowed | Divorced/widowed | 3749 | 1 | 0.41 | 2.43 | 0 | 0.45 | 0.89 | 2.44 |
|  | Married | 16 | Gari, S(2013) | widowed | Divorced/widowed | 1716 | 1.53 | 1.15 | 2.05 | 0.43 | 0.15 | 0.71 | 2.04 |
|  | Married monogamously | 13 | Isingo, R(2012) | Divorced/widowed | Divorced/widowed | 9457 | 1.57 | 1.24 | 2 | 0.45 | 0.12 | 0.69 | 1.99 |
|  | Married | 10 | Teklehaimanot, H. D(2016) | Divorced/widowed | Divorced/widowed | 11919 | 1.03 | 0.47 | 2.26 | 0.03 | 0.40 | 0.81 | 2.26 |
|  | Married | 1 | Paulin, H. N(2015) | Divorced/widowed | Divorced/widowed | 3708 | 0.8 | 0.41 | 1.55 | -0.22 | 0.34 | 0.45 | 1.56 |
|  |  |  |  |  |  |  |  |  |  |  |  |  |  |
| 6 | 20 pts (ref) | 1 | Paulin, H. N(2015) | high level of stigma (80pt/100) | High stigma | 3708 | 0.41 | 0.23 | 0.71 | -0.89 | 0.29 | -0.31 | 0.73 |
|  | No stigma | 9 | Leta, T. H.(2012) | high stigma | High stigma | 6778 | 3.6 | 1.04 | 12.46 | 1.28 | 0.63 | 2.52 | 12.46 |
|  | No stigma | 10 | Teklehaimanot, H. D(2016) | high stigma | High stigma | 11919 | 0.56 | 0.38 | 0.8 | -0.58 | 0.19 | -0.19 | 0.82 |
|  | No | 11 | Tsegay, G(2013) | perceived stigma(yes) | High stigma | 711 | 0.013 | 0.007 | 0.024 | -4.34 | 0.32 | -3.72 | 0.02 |
|  | No | 12 | Kuehne, A.(2018) | Practice of stigma(treat people the same) | High stigma | 9457 | 1.63 | 1.3 | 2.03 | 0.49 | 0.12 | 0.71 | 2.04 |
|  | 50pts | 28 | Mukolo, A | 100pt | High stigma | 2749 | 0.88 | 0.52 | 1.47 | -0.13 | 0.27 | 0.39 | 1.49 |
|  |  |  |  |  |  |  |  |  |  |  |  |  |  |
| 7 | Both(man and woman) | 1 | Paulin, H. N(2015) | Woman | Female sex | 3708 | 1.08 | 0.75 | 1.56 | 0.08 | 0.19 | 0.44 | 1.56 |
|  | Male | 2 | Feleke, S. A(2013) | Female | Female sex | 1290 | 0.38 | 0.26 | 0.56 | -0.96 | 0.19 | -0.58 | 0.56 |
|  | Male | 10 | Teklehaimanot, H. D(2016) | Female | Female sex | 11919 | 0.90 | 0.65 | 1.25 | -0.10 | 0.17 | 0.22 | 1.25 |
|  | Male | 3 | Naidoo, S.(2015) | Female | Female sex | 1114 | 44.9 | 7.77 | 259.38 | 3.80 | 0.89 | 5.56 | 259.46 |
|  | Male | 5 | Schaefer, R, (2017) | Female | Female sex | 8092 | 1.19 | 1.05 | 1.35 | 0.17 | 0.06 | 0.29 | 1.35 |
|  | Male | 27 | Daniyam, C. A.(2010) | Female | Female sex | 368 | 0.63 | 0.31 | 1.26 | -0.46 | 0.36 | 0.25 | 1.28 |
|  | Male | 24 | Apanga, P. A(2015) | Female | Female sex | 200 | 1.1 | 0.39 | 2.9 | 0.09 | 0.53 | 1.13 | 3.10 |
|  | Male | 23 | Sisay, S(2014) | Female | Female sex | 339 | 0.91 | 0.55 | 1.43 | -0.09 | 0.25 | 0.39 | 1.48 |
|  | Male | 22 | Abiodun, O(2014) | Female | Female sex | 1250 | 0.867 | 0.677 | 1.11 | -0.14 | 0.13 | 0.10 | 1.11 |
|  | Male | 20 | Asaolu, Ibitola O(2016) | Female | Female sex | 23367 | 3.125 | 2.70 | 3.57 | 1.14 | 0.07 | 1.29 | 3.61 |
|  | Male | 18 | Bowen, P.(2015) | Female | Female sex | 512 | 4.45 | 1.25 | 15.82 | 1.49 | 0.65 | 2.76 | 15.84 |
|  | Men | 16 | Gari, S(2013) | Women | Female sex | 1716 | 0.67 | 0.5 | 0.75 | -0.40 | 0.15 | -0.11 | 0.89 |
|  | Male | 15 | Haddison, E. C.(2012) | Female | Female sex | 474 | 3.33 | 1.92 | 5 | 1.20 | 0.28 | 1.75 | 5.78 |
|  | Men | 12 | Kuehne, A.(2018) | Women | Female sex | 9457 | 1.56 | 1.29 | 1.88 | 0.44 | 0.09 | 0.63 | 1.88 |
|  |  |  |  |  |  |  |  |  |  |  |  |  |  |
| 8 | < 1 USD/day | 1 | Paulin, H. N(2015) | (> ≈ $1.00 USD/day) | Higher wealth index | 3708 | 1.37 | 0.9 | 2.08 | 0.31 | 0.21 | 0.73 | 2.09 |
|  | Poor | 5 | Schaefer, R, (2017) | Intermediate/less more | Higher wealth index | 8092 | 2.04 | 1.66 | 2.5 | 0.71 | 0.10 | 0.92 | 2.49 |
|  | Lower | 9 | Leta, T. H.(2012) | Higher | Higher wealth index | 6778 | 2.2 | 0.82 | 5.94 | 0.79 | 0.50 | 1.78 | 5.90 |
|  | Low SES | 10 | Teklehaimanot, H. D(2016) | High SES | Higher wealth index | 11919 | 1.3 | 0.73 | 2.29 | 0.26 | 0.29 | 0.84 | 2.32 |
|  | Poor | 16 | Gari, S(2013) | Not poor | Higher wealth index | 1716 | 0.85 | 0.75 | 0.98 | -0.16 | 0.07 | -0.03 | 0.97 |
|  | No health insurance | 12 | Kuehne, A.(2018) | Have health insurance | Higher wealth index | 9457 | 1.1 | 0.84 | 1.48 | 0.09 | 0.14 | 0.36 | 1.44 |
|  |  |  |  |  |  |  |  |  |  |  |  |  |  |
| 9 | Rural | 1 | Paulin, H. N(2015) | Urban | Urban residence | 3708 | 1.36 | 0.91 | 2.04 | 0.31 | 0.20 | 0.71 | 2.03 |
|  | Rural | 5 | Schaefer, R, (2017) | Urban | Urban residence | 8092 | 0.06 | 0.04 | 0.08 | -2.81 | 0.21 | -2.41 | 0.09 |
|  | Rural | 16 | Gari, S(2013) | Urban | Urban residence | 1716 | 2.4 | 1.94 | 2.96 | 0.87 | 0.11 | 1.08 | 2.97 |
|  | Rural | 13 | Isingo, R(2012) | Urban | Urban residence | 9457 | 1.13 | 1.08 | 1.58 | 0.12 | 0.02 | 0.17 | 1.18 |
|  | Isolated district | 28 | Mukolo, A | Urban | Urban residence | 3749 | 1.47 | 1.01 | 2.13 | 0.39 | 0.19 | 0.76 | 2.14 |
|  |  |  |  |  |  |  |  |  |  |  |  |  |  |
| 10 | More access | 1 | Paulin, H. N(2015) | Less access | Less physical access to VCT | 3708 | 1 | 0.97 | 1.03 | 0 | 0.02 | 0.03 | 1.03 |
|  | <3km | 5 | Schaefer, R, (2017) | >3km | Less physical access to VCT | 8092 | 0.83 | 0.74 | 0.94 | -0.18 | 0.06 | -0.07 | 0.93 |
|  | <10mins | 10 | Teklehaimanot, H. D(2016) | 30mins+ | Less physical access to VCT | 11919 | 0.76 | 0.56 | 1.03 | -0.27 | 0.16 | 0.03 | 1.03 |
|  | >5years in germany | 12 | Kuehne, A.(2018) | 1-5years | Less physical access to VCT | 8457 | 1.23 | 0.96 | 1.6 | 0.21 | 0.13 | 0.45 | 1.58 |
|  | more accessible and acceptible | 6 | Motshabi, L. C.(2011) | Poor health system | Less physical access to VCT | 200 | 0.34 | 0.23 | 0.5 | -1.08 | 0.19 | -0.68 | 0.50 |
|  | Accessed health facility | 28 | Mukolo, A.(2013) | Less access | Less physical access to VCT | 3749 | 0.59 | 0.46 | 0.79 | -0.51 | 0.14 | -0.24 | 0.78 |
|  |  |  |  |  |  |  |  |  |  |  |  |  |  |
| 11 | Christian | 1 | Paulin, H. N(2015) | Islam | Islam and other religion | 3708 | 1.02 | 0.59 | 1.76 | 0.02 | 0.28 | 0.57 | 1.76 |
|  | Christian | 24 | Apanga, P. A(2015) | others | Islam and other religion | 200 | 1.11 | 0.42 | 5.55 | 0.11 | 0.49 | 1.08 | 2.94 |
|  | Christian | 22 | Abiodun, O(2014) | others | Islam and other religion | 1250 | 1.35 | 0.83 | 2.18 | 0.29 | 0.25 | 0.78 | 2.19 |
|  | Christian | 19 | Baisley, K.(2012) | Islam | Islam and other religion | 12590 | 0.77 | 0.51 | 1.16 | -0.26 | 0.21 | 0.15 | 1.16 |
|  | Christian | 14 | Huchko, M. J.(2011) | Others | Islam and other religion | 1655 | 0.77 | 0.4 | 1.67 | -0.26 | 0.33 | 0.39 | 1.48 |
|  | Christian | 13 | Isingo, R(2012) | Islam | Islam and other religion | 9457 | 1.09 | 0.72 | 1.65 | 0.09 | 0.21 | 0.50 | 1.65 |
|  | Christian | 12 | Kuehne, A.(2018) | Islam | Islam and other religion | 9457 | 0.66 | 0.53 | 0.81 | -0.42 | 0.11 | -0.19 | 0.82 |
|  | Catholic | 28 | Mukolo, A | Islam | Islam and other religion | 3749 | 0.9 | 0.59 | 1.35 | -0.11 | 0.22 | 0.32 | 1.37 |
|  |  |  |  |  |  |  |  |  |  |  |  |  |  |
| 12 | No | 2 | Feleke, S. A(2013) | Yes (with parents and relative) | Discussion of HIV | 1290 | 1.23 | 0.93 | 1.63 | 0.21 | 0.14 | 0.49 | 1.63 |
|  | No | 10 | Teklehaimanot, H. D(2016) | Talked to partner about HIV | Discussion of HIV | 11919 | 2.06 | 1.61 | 2.71 | 0.72 | 0.13 | 0.97 | 2.64 |
|  | No | 21 | Ajayi, A. I.(2019) | Discuss | Discussion of HIV | 833 | 3.81 | 2.44 | 5.96 | 1.34 | 0.23 | 1.78 | 5.95 |
|  | No | 22 | Abiodun, O(2014) | Media discussion on HIV | Discussion of HIV | 1250 | 1.077 | 0.814 | 1.424 | 0.07 | 0.14 | 0.35 | 1.42 |
|  |  |  |  |  |  |  |  |  |  |  |  |  |  |
| 13 | No | 2 | Feleke, S. A(2013) | Yes | Perceived At-Risk | 1290 | 30 | 10.65 | 83.01 | 3.40 | 0.53 | 4.44 | 84.51 |
|  | No risk | 9 | Leta, T. H.(2012) | High risk | Perceived At-Risk | 6779 | 3 | 1.68 | 5.51 | 1.09 | 0.29 | 1.68 | 5.36 |
|  | No risk | 10 | Teklehaimanot, H. D(2016) | High risk | Perceived At-Risk | 11919 | 1.26 | 0.86 | 1.84 | 0.23 | 0.19 | 0.61 | 1.85 |
|  | Know partner status | 26 | Conserve, D.(2013) | Unknown | Perceived At-Risk | 9107 | 0.22 | 0.19 | 0.25 | -1.51 | 0.05 | -1.41 | 0.25 |
|  | No | 25 | Obermeyer, C. M(2013) | Yes | Perceived At-Risk | 336 | 1.03 | 0.94 | 1.13 | 0.03 | 0.05 | 0.12 | 1.13 |
|  | Low | 23 | Sisay, S(2014) | High | Perceived At-Risk | 339 | 1 | 0.25 | 3.33 | 0 | 0.71 | 1.39 | 4.02 |
|  | Know partner status | 21 | Ajayi, A. I.(2019) | Unknown | Perceived At-Risk | 833 | 0.33 | 0.20 | 0.53 | -1.12 | 0.25 | -0.64 | 0.53 |
|  | No casual partners | 19 | Baisley, K.(2012) | Yes (casual partners in the past 12 months) | Perceived At-Risk | 12590 | 1.52 | 0.96 | 2.38 | 0.41 | 0.23 | 0.88 | 2.41 |
|  | No | 15 | Haddison, E. C.(2012) | Yes | Perceived At-Risk | 474 | 1.21 | 0.68 | 2.14 | 0.19 | 0.29 | 0.77 | 2.15 |
|  | No sex with high risk person | 13 | Isingo, R(2012) | Sex with high risk person without condom | Perceived At-Risk | 9457 | 0.96 | 0.74 | 1.25 | -0.04 | 0.13 | 0.22 | 1.25 |
|  | No | 11 | Tsegay, G(2013) | Yes | Perceived At-Risk | 9457 | 2.4 | 1.37 | 4.23 | 0.88 | 0.29 | 1.44 | 4.20 |
|  | Sex with steady partner | 12 | Kuehne, A.(2018) | Sex with varied partners/inconsistent condom use | Perceived At-Risk | 9457 | 0.72 | 0.56 | 0.92 | -0.33 | 0.13 | -0.07 | 0.93 |
|  |  |  |  |  |  |  |  |  |  |  |  |  |  |
| 14 | Non regular | 3 | Naidoo, S.(2015) | Regular | Condom use | 1114 | 0.36 | 0.13 | 0.99 | -1.01 | 0.51 | -0.01 | 0.98 |
|  | no use | 5 | Schaefer, R, (2017) | use in the last six month | Condom use | 8092 | 1.48 | 1.18 | 1.86 | 0.39 | 0.12 | 0.62 | 1.85 |
|  | No consistent | 26 | Conserve, D.(2013) | consistent use | Condom use | 9107 | 2.12 | 1.77 | 2.55 | 0.75 | 0.09 | 0.93 | 2.54 |
|  | No | 23 | Sisay, S(2014) | yes | Condom use | 339 | 4.5 | 1.1 | 23.3 | 1.50 | 0.72 | 2.91 | 18.41 |
|  | NO | 18 | Bowen, P.(2015) | previously used(yes) | Condom use | 512 | 1.96 | 1.02 | 3.65 | 0.67 | 0.33 | 1.33 | 3.76 |
|  | No consistent | 17 | Dillnessa, E(2010) | regular condom | Condom use | 771 | 4.5 | 1.33 | 15.31 | 1.50 | 0.62 | 2.72 | 15.23 |
|  |  |  |  |  |  |  |  |  |  |  |  |  |  |
| 15 | One or none | 5 | Schaefer, R, (2017) | more than one | Multiple sexual partners | 8092 | 1.22 | 0.87 | 1.71 | 0.19 | 0.17 | 0.54 | 1.71 |
|  | Less | 26 | Conserve, D.(2013) | more | Multiple sexual partners | 9107 | 0.54 | 0.43 | 0.67 | -0.62 | 0.12 | -0.39 | 0.68 |
|  | None | 20 | Asaolu, Ibitola O(2016) | one or more | Multiple sexual partners |  | 0.46 | 0.39 | 0.54 | -0.77 | 0.08 | -0.61 | 0.54 |
|  | Only one | 19 | Baisley, K.(2012) | many | Multiple sexual partners | 12590 | 0.84 | 0.57 | 1.23 | -0.17 | 0.19 | 0.22 | 1.24 |
|  |  | 13 | Isingo, R(2012) | 3+ sexual partners | Multiple sexual partners | 9457 | 3.98 | 2.26 | 7.01 | 1.38 | 0.29 | 1.94 | 7.01 |
|  |  |  |  |  |  |  |  |  |  |  |  |  |  |
|  | No | 21 | Ajayi, A. I.(2019) | received in the past | Attitude/Feeling about VCT | 833 | 2.66 | 1.77 | 3.99 | 0.98 | 0.21 | 1.39 | 3.99 |
|  | No | 15 | Haddison, E. C.(2012) | Should every one receive VCT(yes) | Attitude/Feeling about VCT | 474 | 2.07 | 0.99 | 4.34 | 0.73 | 0.38 | 1.47 | 4.33 |
|  | No | 3 | Naidoo, S.(2015) | Knowing a person who had tested for HIV | Attitude/Feeling about VCT | 1114 | 15.28 | 5.16 | 45.23 | 2.73 | 0.55 | 3.81 | 45.25 |
|  | Confidentiality not assured | 11 | Tsegay, G(2013) | Confidentiality | Attitude/Feeling about VCT | 711 | 3 | 1.65 | 5.49 | 1.09 | 0.31 | 1.69 | 5.45 |
|  | Never tested | 28 | Mukolo, A | Ever tested and test results received | Attitude/Feeling about VCT | 3749 | 7.61 | 5.08 | 11.4 | 2.03 | 0.21 | 2.44 | 11.40 |
|  |  |  |  |  |  |  |  |  |  |  |  |  |  |
| 19 | Yes | 20 | Asaolu, Ibitola O(2016) | Yes | Infection (STI) | 23367 | 1.7 | 1.29 | 2.24 | 0.53 | 0.14 | 0.81 | 2.24 |
|  | No | 13 | Isingo, R(2012) | Infection | Infection (STI) | 9457 | 1.47 | 1.17 | 1.84 | 0.39 | 0.12 | 0.61 | 1.85 |
|  | No | 12 | Kuehne, A.(2018) | Yes | Infection (STI) | 9457 | 2.14 | 1.54 | 2.97 | 0.76 | 0.17 | 1.09 | 2.98 |
|  | No | 4 | Mulogo, E. M(2011) | yes(Sores on penis) | Infection (STI) | 994 | 0.9 | 0.3 | 2.7 | -0.12 | 0.56 | 0.99 | 2.70 |
|  | Negative | 19 | Baisley, K.(2012) | Positive | Infection (STI) | 12590 | 0.86 | 0.66 | 1.14 | -0.15 | 0.14 | 0.11 | 1.12 |
| Ref. No= reference number | | | | | | | | | | | | | |

**Table S4: Summary of categorical determinants of VCT service uptake in SSA**

| **Study** | **Subgroup** | **logOR** | **selogOR** |
| --- | --- | --- | --- |
| Paulin, H. N(2015) | Older age | -0.139761942 | 0.220008982 |
| Feleke, S. A(2013) | Older age | 0.783901544 | 0.129221068 |
| Leta, T. H.(2012) | Older age | -0.405465108 | 0.292276028 |
| Teklehaimanot, H. D(2016) | Older age | 0.609765572 | 0.19319074 |
| Conserve, D.(2013) | Older age | 0.444685821 | 0.066262797 |
| Apanga, P. A(2015) | Older age | -0.223143551 | 0.821141792 |
| Sisay, S(2014) | Older age | 0.693147181 | 0.487505839 |
| Abiodun, O(2014) | Older age | 0.204567166 | 0.152534613 |
| Asaolu, Ibitola O(2016) | Older age | 0.783901544 | 0.048860666 |
| Haddison, E. C.(2012) | Older age | 1.463255402 | 0.789100516 |
| Huchko, M. J.(2011) | Older age | 0.641853886 | 0.23445527 |
| Isingo, R(2012) | Older age | 0.104360015 | 0.107000271 |
| Mukolo, A | Older age | -0.673344553 | 0.237800883 |
|  |  |  |  |
| Paulin, H. N(2015) | Higher education level | 0.21511138 | 0.174971812 |
| Feleke, S. A(2013) | Higher education level | 0.802001585 | 0.259863251 |
| Teklehaimanot, H. D(2016) | Higher education level | 0.862889955 | 0.169521278 |
| Schaefer, R, (2017) | Higher education level | 0.116533816 | 0.07453705 |
| Conserve, D.(2013) | Higher education level | 0.385262401 | 0.176551881 |
| Apanga, P. A(2015) | Higher education level | 1.223775432 | 0.275856422 |
| Sisay, S(2014) | Higher education level | -0.182321557 | 0.285518259 |
| Baisley, K.(2012) | Higher education level | -0.438254931 | 0.176350313 |
| Dillnessa, E(2010) | Higher education level | 1.355835154 | 0.59029811 |
| Gari, S(2013) | Higher education level | -0.030459207 | 0.451954859 |
| Haddison, E. C.(2012) | Higher education level | 0.148420005 | 0.243328608 |
| Huchko, M. J.(2011) | Higher education level | 1.280933845 | 0.299891156 |
| Isingo, R(2012) | Higher education level | 0.819779831 | 0.172119226 |
| Kuehne, A.(2018) | Higher education level | 0.542324291 | 0.127374835 |
|  |  |  |  |
| Leta, T. H.(2012) | Higher knowledge of HIV/VCT | -0.356674944 | 0.285518259 |
| Abiodun, O(2014) | Higher knowledge of HIV/VCT | 0.102556588 | 0.38107837 |
| Teklehaimanot, H. D(2016) | Higher knowledge of HIV/VCT | 0.672944473 | 0.237719543 |
| Asaolu, Ibitola O(2016) | Higher knowledge of HIV/VCT | 0.683096845 | 0.060093386 |
| Baisley, K.(2012) | Higher knowledge of HIV/VCT | -0.261364764 | 0.153445997 |
| Tsegay, G(2013) | Higher knowledge of HIV/VCT | 1.305626458 | 0.577384261 |
| Kuehne, A.(2018) | Higher knowledge of HIV/VCT | 0.418710335 | 0.095713579 |
| Mukolo, A | Higher knowledge of HIV/VCT | 0.518793793 | 0.234961676 |
|  |  |  |  |
| Paulin, H. N(2015) | Being married | 0.300104592 | 0.163422092 |
| Leta, T. H.(2012) | Being married | 0.356674944 | 0.307926032 |
| Teklehaimanot, H. D(2016) | Being married | -0.274436846 | 0.174361885 |
| Conserve, D.(2013) | Being married | -0.593326845 | 0.075821683 |
| Apanga, P. A(2015) | Being married | -0.09531018 | 0.456240313 |
| Baisley, K.(2012) | Being married | 0.040821995 | 0.074072454 |
| Gari, S(2013) | Being married | 0.916290732 | 0.103541247 |
| Huchko, M. J.(2011) | Being married | -0.356674944 | 0.171669508 |
| Isingo, R(2012) | Being married | -0.010050336 | 0.141648845 |
|  |  |  |  |
| Mukolo, A | Divorced/widowed | 0 | 0.454897 |
| Gari, S(2013) | Divorced/widowed | 0.425267735 | 0.145666221 |
| Isingo, R(2012) | Divorced/widowed | 0.451075619 | 0.120389918 |
| Teklehaimanot, H. D(2016) | Divorced/widowed | 0.029558802 | 0.400296626 |
| Paulin, H. N(2015) | Divorced/widowed | -0.223143551 | 0.341048249 |
|  |  |  |  |
| Paulin, H. N(2015) | High stigma | -0.891598119 | 0.294937679 |
| Leta, T. H.(2012) | High stigma | 1.280933845 | 0.633527108 |
| Teklehaimanot, H. D(2016) | High stigma | -0.579818495 | 0.197839557 |
| Tsegay, G(2013) | High stigma | -4.342805922 | 0.315836331 |
| Kuehne, A.(2018) | High stigma | 0.488580015 | 0.115416199 |
| Mukolo, A | High stigma | -0.127833372 | 0.268414845 |
|  |  |  |  |
| Paulin, H. N(2015) | Female sex | 0.076961041 | 0.186042405 |
| Feleke, S. A(2013) | Female sex | -0.955511445 | 0.193617154 |
| Teklehaimanot, H. D(2016) | Female sex | -0.104360015 | 0.167052245 |
| Naidoo, S.(2015) | Female sex | 3.804437795 | 0.894983485 |
| Schaefer, R, (2017) | Female sex | 0.173953307 | 0.063858746 |
| Daniyam, C. A.(2010) | Female sex | -0.46203546 | 0.36180996 |
| Apanga, P. A(2015) | Female sex | 0.09531018 | 0.529040163 |
| Sisay, S(2014) | Female sex | -0.09531018 | 0.251263513 |
| Abiodun, O(2014) | Female sex | -0.142716302 | 0.126208012 |
| Asaolu, Ibitola O(2016) | Female sex | 1.139434283 | 0.074072454 |
| Bowen, P.(2015) | Female sex | 1.492904096 | 0.647837013 |
| Gari, S (2013) | Female sex | -0.400477567 | 0.149321232 |
| Haddison, E. C.(2012) | Female sex | 1.202972304 | 0.280942407 |
| Kuehne, A.(2018) | Female sex | 0.444685821 | 0.096961022 |
|  |  |  |  |
| Paulin, H. N(2015) | Higher wealth index | 0.31481074 | 0.21437309 |
| Schaefer, R, (2017) | Higher wealth index | 0.713349888 | 0.103328706 |
| Leta, T. H.(2012) | Higher wealth index | 0.78845736 | 0.503524642 |
| Teklehaimanot, H. D (2016) | Higher wealth index | 0.262364264 | 0.294426025 |
| Gari, S(2013) | Higher wealth index | -0.157003749 | 0.065395507 |
| Kuehne, A. (2018) | Higher wealth index | 0.09531018 | 0.137583453 |
|  |  |  |  |
| Paulin, H. N (2015) | Urban residence | 0.3074847 | 0.204997642 |
| Schaefer, R, (2017) | Urban residence | -2.813410717 | 0.206869953 |
| Gari, S (2013) | Urban residence | 0.875468737 | 0.108561614 |
| Isingo, R (2012) | Urban residence | 0.122217633 | 0.023090098 |
| Mukolo, A | Urban residence | 0.385662481 | 0.191638849 |
|  |  |  |  |
| Paulin, H. N(2015) | more physical access to VC | 0 | 0.015081022 |
| Schaefer, R, (2017) | more physical access to VC | -0.186329578 | 0.058558936 |
| Teklehaimanot, H. D(2016) | more physical access to VC | -0.274436846 | 0.155806964 |
| Kuehne, A.(2018) | more physical access to VC | 0.207014169 | 0.126447022 |
| Motshabi, L. C.(2011) | more physical access to VC | -1.078809661 | 0.199421586 |
| Mukolo, A.(2013) | more physical access to VC | -0.512823626 | 0.13830506 |
|  |  |  |  |
| Paulin, H. N(2015) | Islam and other religion | 0.019802627 | 0.27930376 |
| Apanga, P. A(2015) | Islam and other religion | 0.105360516 | 0.496153573 |
| Abiodun, O(2014) | Islam and other religion | 0.299754654 | 0.246382243 |
| Baisley, K.(2012) | Islam and other religion | -0.261364764 | 0.21019377 |
| Huchko, M. J.(2011) | Islam and other religion | -0.262364264 | 0.333635953 |
| Isingo, R(2012) | Islam and other religion | 0.086177696 | 0.211572328 |
| Kuehne, A.(2018) | Islam and other religion | -0.415515444 | 0.11191981 |
| Mukolo, A | Islam and other religion | -0.105360516 | 0.215445013 |
|  |  |  |  |
| Feleke, S. A(2013) | Discussion of HIV | 0.207014169 | 0.142645338 |
| Teklehaimanot, H. D(2016) | Discussion of HIV | 0.722705983 | 0.12575092 |
| Ajayi, A. I.(2019) | Discussion of HIV | 1.337629189 | 0.227362832 |
| Abiodun, O(2014) | Discussion of HIV | 0.074179398 | 0.142844036 |
|  |  |  |  |
| Feleke, S. A (2013) | Perceived At-Risk | 3.401197382 | 0.528386474 |
| Leta, T. H. (2012) | Perceived At-Risk | 1.098612289 | 0.295825763 |
| Teklehaimanot, H. D (2016) | Perceived At-Risk | 0.231111721 | 0.194864597 |
| Conserve, D.(2013) | Perceived At-Risk | -1.510721939 | 0.05341737 |
| Obermeyer, C. M (2013) | Perceived At-Risk | 0.03 | 0.045918367 |
| Sisay, S (2014) | Perceived At-Risk | 0 | 0.709837705 |
| Ajayi, A. I. (2019) | Perceived At-Risk | -1.121677562 | 0.245786877 |
| Baisley, K. (2012) | Perceived At-Risk | 0.418710335 | 0.23445527 |
| Haddison, E. C.(2012) | Perceived At-Risk | 0.19062036 | 0.294021857 |
| Isingo, R (2012) | Perceived At-Risk | -0.040821995 | 0.132797499 |
| Tsegay, G (2013) | Perceived At-Risk | 0.875468737 | 0.286049999 |
| Kuehne, A. (2018) | Perceived At-Risk | -0.328504067 | 0.128221647 |
|  |  |  |  |
| Naidoo, S.(2015) | Condom use | -1.011600912 | 0.509843922 |
| Schaefer, R, (2017) | Condom use | 0.392042088 | 0.115575331 |
| Conserve, D.(2013) | Condom use | 0.751416089 | 0.09205946 |
| Sisay, S(2014) | Condom use | 1.504077397 | 0.718758784 |
| Bowen, P. (2015) | Condom use | 0.672944473 | 0.333235636 |
| Dillnessa, E (2010) | Condom use | 1.504077397 | 0.621886967 |
|  |  |  |  |
| Schaefer, R, (2017) | Multiple sexual partners | 0.198850859 | 0.172506595 |
| Conserve, D.(2013) | Multiple sexual partners | -0.616186139 | 0.116216291 |
| Asaolu, Ibitola O(2016) | Multiple sexual partners | -0.776528789 | 0.084224362 |
| Baisley, K.(2012) | Multiple sexual partners | -0.174353387 | 0.197839557 |
| Isingo, R(2012) | Multiple sexual partners | 1.381281819 | 0.288733166 |
|  |  |  |  |
| Ajayi, A. I. (2019) | Attitude/Feeling about VCT | 0.978326123 | 0.207829886 |
| Haddison, E. C.(2012) | Attitude/Feeling about VCT | 0.727548607 | 0.376325991 |
| Naidoo, S.(2015) | Attitude/Feeling about VCT | 2.726544784 | 0.553881737 |
| Tsegay, G(2013) | Attitude/Feeling about VCT | 1.098612289 | 0.305018878 |
| Mukolo, A | Attitude/Feeling about VCT | 2.029463172 | 0.206199954 |
|  |  |  |  |
| Asaolu, Ibitola O(2016) | Infection (STI) | 0.530628251 | 0.1408092 |
| Isingo, R(2012) | Infection (STI) | 0.385262401 | 0.116458496 |
| Kuehne, A.(2018) | Infection (STI) | 0.760805829 | 0.167869088 |
| Mulogo, E. M(2011) | Infection (STI) | -0.105360516 | 0.560516474 |
| Baisley, K.(2012) | Infection (STI) | -0.15082289 | 0.135047222 |

**File S1. Meta-regression results**

**Meta-regression for “Older age subgroup”**

metareg(meta2, ~Age_cat, hakn = T)

Mixed-Effects Model (k = 13; tau^2 estimator: DL)

Estimated amount of residual heterogeneity: tau^2 = 0.1403 (SE = 0.1025)

Square root of estimated tau^2 value: tau = 0.3745

Residual heterogeneity / unaccounted variability: I^2= 85.52%

Unaccounted variability / sampling variability: H^2= 6.90

Amount of heterogeneity accounted for: R^2 = 0.00%

Test for Residual Heterogeneity:

QE (df = 11) = 75.9495, p-val < .0001

Test of Moderators (coefficient 2):

F (df1 = 1, df2 = 11) = 9.7389, p-val = 0.0097

**Model Results**:

| Age_category | Estimate | se | t-val | p-val | ci.lb | ci.ub |  |
| --- | --- | --- | --- | --- | --- | --- | --- |
| intrcpt | 0.5614 | 0.1344 | 4.1769 | 0.0015 | 0.2656 | 0.8571 | ** |
| Age_cat>30 | -0.6990 | 0.2240 | -3.1207 | 0.0097 | -1.1919 | -0.2060 | ** |

Signif. codes: 0 ‘***’ 0.001 ‘**’ 0.01 ‘*’ 0.05 ‘.’ 0.1 ‘ ’ 1

**metareg(meta3, ~Age_cat+S.SIZE, hakn = T)**

Mixed-Effects Model (k = 13; tau^2 estimator: DL)

Estimated amount of residual heterogeneity: tau^2 =0.1464 (SE = 0.1116)

Square root of estimated tau^2 value: tau =0.3826

Residual heterogeneity / unaccounted variability: I^2 =84.75%

Unaccounted variability / sampling variability: H^2 =6.56

Amount of heterogeneity accounted for: R^2 =0.00%

Test for Residual Heterogeneity:

QE (df = 10) = 65.5921, p-val < .0001

Test of Moderators (coefficients 2:3):

F (df1 = 2, df2 = 10) = 4.7911, p-val = 0.0347

**Model Results:**

| Age&S.size | Estimate | se | t-val | p-val | ci.lb | ci.ub |  |
| --- | --- | --- | --- | --- | --- | --- | --- |
| intrcpt | 0.4844 | 0.1927 | 2.5142 | 0.0307 | 0.0551 | 0.9137 | * |
| Age_cat>30 | -0.6887 | 0.2321 | -2.9667 | 0.0141 | -1.2060 | -0.1715 | * |
| S.SIZE>5000 | 0.1309 | 0.2245 | 0.5832 | 0.5727 | -0.3693 | 0.6311 |  |

Signif. codes: 0 ‘***’ 0.001 ‘**’ 0.01 ‘*’ 0.05 ‘.’ 0.1 ‘ ’ 1

**Meta-regression for “female subgroup”**

1. met_reg.sex.1<-metareg(met, ~Publication_date, intercept = F)

Mixed-Effects Model (k = 14; tau^2 estimator: DL)

Estimated amount of residual heterogeneity tau^2 =0.2978 (SE = 0.2047)

Square root of estimated tau^2 value: tau =0.5458

Residual heterogeneity / unaccounted variability: I^2 =93.27%

Unaccounted variability / sampling variability: H^2 =14.85

Test for Residual Heterogeneity:

QE (df = 12) = 178.2191, p-val < .0001

Test of Moderators (coefficients 1:2):

F (df1 = 2, df2 = 12) = 1.8834, p-val = 0.1944

Model Results:

| Publication date | estimate | se | tval | pval | ci.lb | ci.ub |  |
| --- | --- | --- | --- | --- | --- | --- | --- |
| =>2015 | 0.5706 | 0.3036 | 1.8798 | 0.0846 | -0.0908 | 1.2320 |  |
| <2015 | -0.1586 | 0.3286 | -0.4827 | 0.6380 | -0.8745 | 0.5573 |  |

Signif. codes: 0 ‘***’ 0.001 ‘**’ 0.01 ‘*’ 0.05 ‘.’ 0.1 ‘ ’ 1

1. met_reg.sex<-metareg(met, ~Location, hakn = T)

**Mixed-Effects Model (k = 14; tau^2 estimator: DL)**

Estimated amount of residual heterogeneity: tau^2 = 0.4513 (SE = 0.3291)

Square root of estimated tau^2 value: tau= 0.6718

Residual heterogeneity / unaccounted variability: I^2 =95.10%

Unaccounted variability / sampling variability: H^2 =20.42

Amount of heterogeneity accounted for: R^2 =0.00%

Test for Residual Heterogeneity:

QE(df = 11) = 224.6022, p-val < .0001

Test of Moderators (coefficients 2:3):

F(df1 = 2, df2 = 11) = 0.0270, p-val = 0.9734

**Model Results:**

| Location | estimate | se | tval | pval | ci.lb | ci.ub |  |
| --- | --- | --- | --- | --- | --- | --- | --- |
| intercept | 0.3664 | 0.5619 | 0.6519 | 0.5278 | -0.8705 | 1.6032 |  |
| Rural &Urban | -0.0915 | 0.7470 | -0.1226 | 0.9047 | -1.7356 | 1.5525 |  |
| Urban | -0.1593 | 0.6892 | -0.2311 | 0.8215 | -1.6761 | 1.3576 |  |

Signif. codes: 0 ‘***’ 0.001 ‘**’ 0.01 ‘*’ 0.05 ‘.’ 0.1 ‘ ’ 1

**Meta-regression for “Education subgroup”**

1. metareg(samboukuka, ~E.level, hakn = T)

Mixed-Effects Model (k = 14; tau^2 estimator: DL)

Estimated amount of residual heterogeneity: tau^2 =0.1633 (SE = 0.1073)

Square root of estimated tau^2 value: tau =0.4042

Residual heterogeneity / unaccounted variability: I^2=81.78%

Unaccounted variability / sampling variability: H^2 =5.49

Amount of heterogeneity accounted for: R^2 =3.35%

Test for Residual Heterogeneity:

QE (df = 11) = 60.3670, p-val < .0001

Test of Moderators (coefficients 2:3):

F (df1 = 2, df2 = 11) = 2.8383, p-val = 0.1014

**Model Results:**

| Education level | Estimate | se | t-val | p-val | ci.lb | ci.ub |  |
| --- | --- | --- | --- | --- | --- | --- | --- |
| Intercept | 0.6321 | 0.2957 | 2.1376 | 0.0558 | -0.0187 | 1.2830 | . |
| Secondary | -0.3947 | 0.3374 | -1.1697 | 0.2668 | -1.1373 | 0.3480 |  |
| Tertiary | 0.3310 | 0.4006 | 0.8262 | 0.4262 | -0.5508 | 1.2128 |  |

Signif. codes: 0 ‘***’ 0.001 ‘**’ 0.01 ‘*’ 0.05 ‘.’ 0.1 ‘ ’ 1

1. **metareg(samboukuka, ~Region, hakn = T)**

Mixed-Effects Model (k = 14; tau^2 estimator: DL)

Estimated amount of residual heterogeneity: tau^2 =0.2082 (SE = 0.1283)

Square root of estimated tau^2 value: tau =0.4563

Residual heterogeneity / unaccounted variability: I^2 =82.78%

Unaccounted variability / sampling variability: H^2 =5.81

Amount of heterogeneity accounted for: R^2 = 0.00%

Test for Residual Heterogeneity:

QE (df = 10) = 58.0741, p-val < .0001

Test of Moderators (coefficients 2:4):

F (df1 = 3, df2 = 10) = 0.9247, p-val = 0.4640

**Model Results:**

| Region | estimate | se | tval | pval | ci.lb | ci.ub |  |
| --- | --- | --- | --- | --- | --- | --- | --- |
| Intercept | 0.1484 | 0.5414 | 0.2742 | 0.7895 | -1.0578 | 1.3546 |  |
| East Africa | 0.3989 | 0.5752 | 0.6935 | 0.5038 | -0.8827 | 1.6805 |  |
| South Africa | -0.0269 | 0.6246 | -0.0431 | 0.9665 | -1.4186 | 1.3648 |  |
| West Africa | 0.6945 | 0.6561 | 1.0585 | 0.3147 | -0.7674 | 2.1565 |  |

Signif. codes: 0 ‘***’ 0.001 ‘**’ 0.01 ‘*’ 0.05 ‘.’ 0.1 ‘ ’ 1

1. **metareg(samboukuka, ~S.size, hakn = T)**

Mixed-Effects Model (k = 14; tau^2 estimator: DL)

Estimated amount of residual heterogeneity: tau^2 =0.1866 (SE = 0.1166)

Square root of estimated tau^2 value: tau =0.4320

Residual heterogeneity / unaccounted variability: I^2 =84.31%

Unaccounted variability / sampling variability: H^2 =6.37

Amount of heterogeneity accounted for: R^2= 0.00%

Test for Residual Heterogeneity:

QE (df = 12) = 76.4896, p-val < .0001

Test of Moderators (coefficient 2):

F (df1 = 1, df2 = 12) = 0.2826, p-val = 0.6047

**Model Results:**

| S.size | estimate | se | tval | pval | ci.lb | ci.ub |  |
| --- | --- | --- | --- | --- | --- | --- | --- |
| intercept | 0.5365 | 0.1941 | 2.7639 | 0.0172 | 0.1136 | 0.9594 | * |
| S. Size>5000 | -0.1587 | 0.2986 | -0.5316 | 0.6047 | -0.8093 | 0.4918 |  |

Signif. codes: 0 ‘***’ 0.001 ‘**’ 0.01 ‘*’ 0.05 ‘.’ 0.1 ‘ ’ 1

1. **metareg(samboukuka, ~Location, hakn = T)**

Mixed-Effects Model (k = 14; tau^2 estimator: DL)

Estimated amount of residual heterogeneity: tau^2 =0.2357 (SE = 0.1319)

Square root of estimated tau^2 value: tau= 0.4855

Residual heterogeneity / unaccounted variability: I^2 =84.13%

Unaccounted variability / sampling variability: H^2 =6.30

Amount of heterogeneity accounted for: R^2 = 0.00%

Test for Residual Heterogeneity:

QE (df = 11) = 69.3070, p-val < .0001

Test of Moderators (coefficients 2:3):

F (df1 = 2, df2 = 11) = 0.3989, p-val = 0.6804

**Model Results:**

| Location | Estimate | se | tval | pval | ci.lb | ci.ub |  |
| --- | --- | --- | --- | --- | --- | --- | --- |
| intercept | 0.3264 | 0.2421 | 1.3484 | 0.2047 | -0.2064 | 0.8593 |  |
| Rural &Urban | 0.1243 | 0.4135 | 0.3007 | 0.7693 | -0.7857 | 1.0343 |  |
| Urban | 0.3026 | 0.3401 | 0.8899 | 0.3926 | -0.4459 | 1.0512 |  |

Signif. codes: 0 ‘***’ 0.001 ‘**’ 0.01 ‘*’ 0.05 ‘.’ 0.1 ‘ ’ 1

1. **metareg(samboukuka, ~Place_of_study, hakn = T)**

Mixed-Effects Model (k = 14; tau^2 estimator: DL)

Estimated amount of residual heterogeneity: tau^2 = 0.1773 (SE = 0.1122)

Square root of estimated tau^2 value: tau =0.4211

Residual heterogeneity / unaccounted variability: I^2 =84.15%

Unaccounted variability / sampling variability: H^2 =6.31

Amount of heterogeneity accounted for: R^2 =0.00%

Test for Residual Heterogeneity:

QE (df = 12) = 75.7016, p-val < .0001

Test of Moderators (coefficient 2):

F (df1 = 1, df2 = 12) = 0.0462, p-val = 0.8334

**Model Results:**

| Place_of_study | estimate | se | tval | pval | ci.lb | ci.ub |  |
| --- | --- | --- | --- | --- | --- | --- | --- |
| intercept | 0.4465 | 0.1803 | 2.4767 | 0.0291 | 0.0537 | 0.8393 | * |
| Institution | 0.0685 | 0.3187 | 0.2150 | 0.8334 | -0.6259 | 0.7630 |  |

Signif. codes: 0 ‘***’ 0.001 ‘**’ 0.01 ‘*’ 0.05 ‘.’ 0.1 ‘ ’ 1

**Combined**

metareg(edu2,~ S.size+ E.level+ Region, hakn = T, intercept = T)

Mixed-Effects Model (k = 14; tau^2 estimator: DL)

Estimated amount of residual heterogeneity: tau^2 =0.4825 (SE = 0.4424)

Square root of estimated tau^2 value: tau=0.6946

Residual heterogeneity / unaccounted variability: I^2 =89.35%

Unaccounted variability / sampling variability: H^2=9.39

Amount of heterogeneity accounted for: R^2= 0.00%

Test for Residual Heterogeneity:

QE(df = 4) = 37.5729, p-val < .0001

Test of Moderators (coefficients 2:10):

F(df1 = 9, df2 = 4) = 0.7714, p-val = 0.6586

Model Results:

| Education subgroup | estimate | se | tval | pval | ci.lb | ci.ub |  |
| --- | --- | --- | --- | --- | --- | --- | --- |
| intrcpt | 0.8829 | 1.1645 | 0.7581 | 0.4906 | -2.3504 | 4.1162 |  |
| S.size>5000 | 0.1318 | 0.4871 | 0.2706 | 0.8001 | -1.2206 | 1.4842 |  |
| E.level: secondary | -0.8511 | 0.6484 | -1.3127 | 0.2595 | -2.6514 | 0.9491 |  |
| E.level: tertiary | 0.3734 | 0.9333 | 0.4000 | 0.7096 | -2.2180 | 2.9647 |  |
| Region: East Africa | 0.2143 | 0.9024 | 0.2374 | 0.8240 | -2.2912 | 2.7197 |  |
| Region: South Africa | -0.6703 | 1.2690 | -0.5283 | 0.6253 | -4.1935 | 2.8528 |  |
| Region: West Africa | -0.6537 | 1.3566 | -0.4818 | 0.6551 | -4.4203 | 3.1129 |  |
| Location: Rural &Urban | 0.6184 | 0.7344 | 0.8420 | 0.4472 | -1.4207 | 2.6576 |  |
| Location: Urban | -0.1896 | 0.5142 | -0.3687 | 0.7311 | -1.6172 | 1.2381 |  |
| Place_of_study: Instittution | 0.1167 | 0.5405 | 0.2158 | 0.8397 | -1.3841 | 1.6174 |  |

Signif. codes: 0 ‘***’ 0.001 ‘**’ 0.01 ‘*’ 0.05 ‘.’ 0.1 ‘ ’ 1

**Meta-regression for ”perceived-at risk subgroup”**

**met_reg.p.1**

Mixed-Effects Model (k = 11; tau^2 estimator: DL)

Estimated amount of residual heterogeneity): tau^2=1.0139 (SE = 0.6383)

Square root of estimated tau^2 value): tau =1.0069

Residual heterogeneity / unaccounted variability: I^2 =95.32%

Unaccounted variability / sampling variability: H^2 =21.38

Test for Residual Heterogeneity:

QE(df = 8) = 171.0468, p-val < .0001

Test of Moderators (coefficients 1:3):

F(df1 = 3, df2 = 8) = 0.1766, p-val = 0.9093

**Model Results:**

| **Location** | estimate | se | tval | pval | ci.lb | ci.ub |  |
| --- | --- | --- | --- | --- | --- | --- | --- |
| Both | 0.5428 | 0.9448 | 0.5745 | 0.5814 | -1.6360 | 2.7215 |  |
| Rural | 0.1979 | 0.6698 | 0.2955 | 0.7751 | -1.3467 | 1.7426 |  |
| Urban | 0.2112 | 0.6297 | 0.3353 | 0.7460 | -1.2410 | 1.6633 |  |

Signif. codes: 0 ‘***’ 0.001 ‘**’ 0.01 ‘*’ 0.05 ‘.’ 0.1 ‘ ’ 1

**> met_reg.p.3**

Mixed-Effects Model (k = 11; tau^2 estimator: DL)

Estimated amount of residual heterogeneity): tau^2 =1.0475 (SE = 1.0409)

Square root of estimated tau^2 value: tau=1.0235

Residual heterogeneity / unaccounted variability: I^2 =98.58%

Unaccounted variability / sampling variability: H^2 =70.27

Test for Residual Heterogeneity:

QE (df = 9) = 632.4034, p-val < .0001

Test of Moderators (coefficients 1:2):

F (df1 = 2, df2 = 9) = 0.5523, p-val = 0.5939

**Model Results:**

| **population type** | estimate | se | tval | pval | ci.lb | ci.ub |  |
| --- | --- | --- | --- | --- | --- | --- | --- |
| others | 0.0203 | 0.4997 | 0.0406 | 0.9685 | -1.1102 | 1.1507 |  |
| Students | 0.6132 | 0.5838 | 1.0502 | 0.3210 | -0.7076 | 1.9339 |  |

Signif. codes: 0 ‘***’ 0.001 ‘**’ 0.01 ‘*’ 0.05 ‘.’ 0.1 ‘ ’ 1

**> met_reg.p.4**

Mixed-Effects Model (k = 11; tau^2 estimator: DL)

Estimated amount of residual heterogeneity: tau^2 =1.1965 (SE = 0.8600)

Square root of estimated tau^2 value: tau =1.0939

Residual heterogeneity / unaccounted variability: I^2 =97.34%

Unaccounted variability / sampling variability: H^2 =37.58

Test for Residual Heterogeneity:

QE (df = 9) = 338.2064, p-val < .0001

Test of Moderators (coefficients 1:2):

F (df1 = 2, df2 = 9) = 0.4599, p-val = 0.6454

**Model Results:**

| **Publication_date** | estimate | se | tval | pval | ci.lb | ci.ub |  |
| --- | --- | --- | --- | --- | --- | --- | --- |
| >5000 | 0.0208 | 0.5580 | 0.0373 | 0.9711 | -1.2416 | 1.2832 |  |
| <5000 | 0.5095 | 0.5316 | 0.9583 | 0.3629 | -0.6931 | 1.7121 |  |

Signif. codes: 0 ‘***’ 0.001 ‘**’ 0.01 ‘*’ 0.05 ‘.’ 0.1 ‘ ’ 1

**> met_reg.p.5**

Mixed-Effects Model (k = 11; tau^2 estimator: DL)

Estimated amount of residual heterogeneity: tau^2=1.1335 (SE = 1.1944)

Square root of estimated tau^2 value: tau =1.0647

Residual heterogeneity / unaccounted variability: I^2 =98.58%

Unaccounted variability / sampling variability: H^2 =70.34

Test for Residual Heterogeneity:

QE(df = 9) = 633.0894, p-val < .0001

Test of Moderators (coefficients 1:2):

F(df1 = 2, df2 = 9) = 0.5009, p-val = 0.6220

**Model Results:**

| **place_study** | estimate | se | tval | pval | ci.lb | ci.ub |  |
| --- | --- | --- | --- | --- | --- | --- | --- |
| Community | 0.5126 | 0.5126 | 0.9999 | 0.3435 | -0.6470 | 1.6722 |  |
| Institution | -0.0248 | 0.5750 | -0.0432 | 0.9665 | -1.3257 | 1.2760 |  |

Signif. codes: 0 ‘***’ 0.001 ‘**’ 0.01 ‘*’ 0.05 ‘.’ 0.1 ‘ ’ 1

**Combined effect**

prr<-metareg(pr, ~population_type+study.place+P.date+S.size, hakn = T, intercept = T)

> prr

Mixed-Effects Model (k = 11; tau^2 estimator: DL)

Estimated amount of residual heterogeneity: tau^2 =2.6766 (SE = 2.1127)

Square root of estimated tau^2 value: tau =1.6360

Residual heterogeneity / unaccounted variability: I^2 =97.91%

Unaccounted variability / sampling variability: H^2 =47.92

Amount of heterogeneity accounted for: R^2 =0.00%

Test for Residual Heterogeneity:

QE(df = 6) = 287.4959, p-val < .0001

Test of Moderators (coefficients 2:5):

F(df1 = 4, df2 = 6) = 1.2300, p-val = 0.3901

**Model Results:**

| **Perceived at-risk** | estimate | se | tval | pval | ci.lb | ci.ub |  |
| --- | --- | --- | --- | --- | --- | --- | --- |
| intrcpt | -0.4208 | 0.9423 | -0.4465 | 0.6709 | -2.7265 | 1.8850 |  |
| population_type: Students | 2.1869 | 1.5248 | 1.4342 | 0.2015 | -1.5442 | 5.9180 |  |
| study.place: Institution | -1.6904 | 0.9559 | -1.7685 | 0.1274 | -4.0294 | 0.6485 |  |
| P.date: before 2015 | 0.9909 | 0.9439 | 1.0497 | 0.3343 | -1.3189 | 3.3006 |  |
| S.size: <5000 | -0.5401 | 1.3222 | -0.4085 | 0.6971 | -3.7753 | 2.6951 |  |

Signif. codes: 0 ‘***’ 0.001 ‘**’ 0.01 ‘*’ 0.05 ‘.’ 0.1 ‘ ’ 1

_______________________________________________________________________________________________________________________________

**Table S5. Results of the 14 categorical determinants of VCT service Uptake in SSA identified from**

| **No.** | **Determinants** | **Studies** | **OR (95%CI)** | *I*^2^(**p-value)** | **Tau^2^** |
| --- | --- | --- | --- | --- | --- |
| 1 | Condom use | (3, 4, 32, 37, 45-50) | **1.75 (1.18-2.58)** | 75(p < 0.01) | 0.1256 |
| 2 | Discussion of HIV | (8, 15, 26, 51) | **1.76 (1.10-2.81)** | 90(p < 0.01) | 0.2054 |
| 3 | Divorced/widowed | (4, 26, 37, 45, 46) | **1.44 (1.22-1.70)** | 20(P=0.29) | 0.0120 |
| 4 | Female sex | (1, 2, 4, 8, 14, 15, 26, 32, 37, 47, 49, 50, 52, 53) | 1.29 (0.90-1.84) | 95(p < 0.01) | 0.3816 |
| 5 | High stigma | (4, 16, 26, 27, 46, 53) | 0.49 (0.14-1.73) | 98(p < 0.01) | 2.4147 |
| 6 | Higher education level | (1, 3, 4, 15, 26, 37, 45, 47, 48, 50, 52-55) | **1.60 (1.24-2.05)** | 84(p < 0.01) | 0.1690 |
| 7 | Higher knowledge of HIV/VCT | (2, 8, 16, 26, 27, 46, 53, 54) | **1.40 (1.03-1.90)** | 85(p < 0.01) | 0.1344 |
| 8 | Higher wealth index | (4, 16, 26, 37, 47, 53) | 1.34 (0.90-1.98) | 91(p < 0.01) | 0.1894 |
| 9 | Infection (STI) | (2, 45, 53, 54, 56) | **1.40 (1.00-1.98)** | 82(p < 0.01) | 0.1122 |
| 10 | Less physical access to VCT | (4, 26, 46, 47, 53, 57) | **0.77 (0.62-0.96)** | 91(p < 0.01) | 0.0586 |
| 11 | Multiple sexual partners | (2, 3, 45, 47, 54) | 0.96 (0.55-1.66) | 95(p < 0.01) | 0.3626 |
| 12 | Older age | (1-4, 8, 15, 16, 26, 45, 46, 50, 52, 55) | **1.36 (1.08-1.73)** | 88(p < 0.01) | 0.1302 |
| 13 | Perceived At−Risk | (3, 6, 15, 16, 26, 27, 50-54) | 1.23 (0.70-2.18) | 98(p < 0.01) | 0.9140 |
| 14 | Urban residence | (4, 37, 45-47) | 0.81 (0.36-1.81) | 98(p < 0.01) | 0.8230 |
| Random effects model: | | Combine OR(CI): 1.20 (1.07-1.33) | | | |
| Heterogeneity: I^2^ = 96%, τ2 = 0.2661, p = 0.01  Residual heterogeneity: I^2^ = 95%, p =0.01  Subgroup differences: χ^2^_13_ = 969.53, df = 13 (p < 0.01) | | | | | |

**Figure S1: Forest plot showing results of the 14 categorical determinants of VCT service Uptake in SSA identified from literature.**


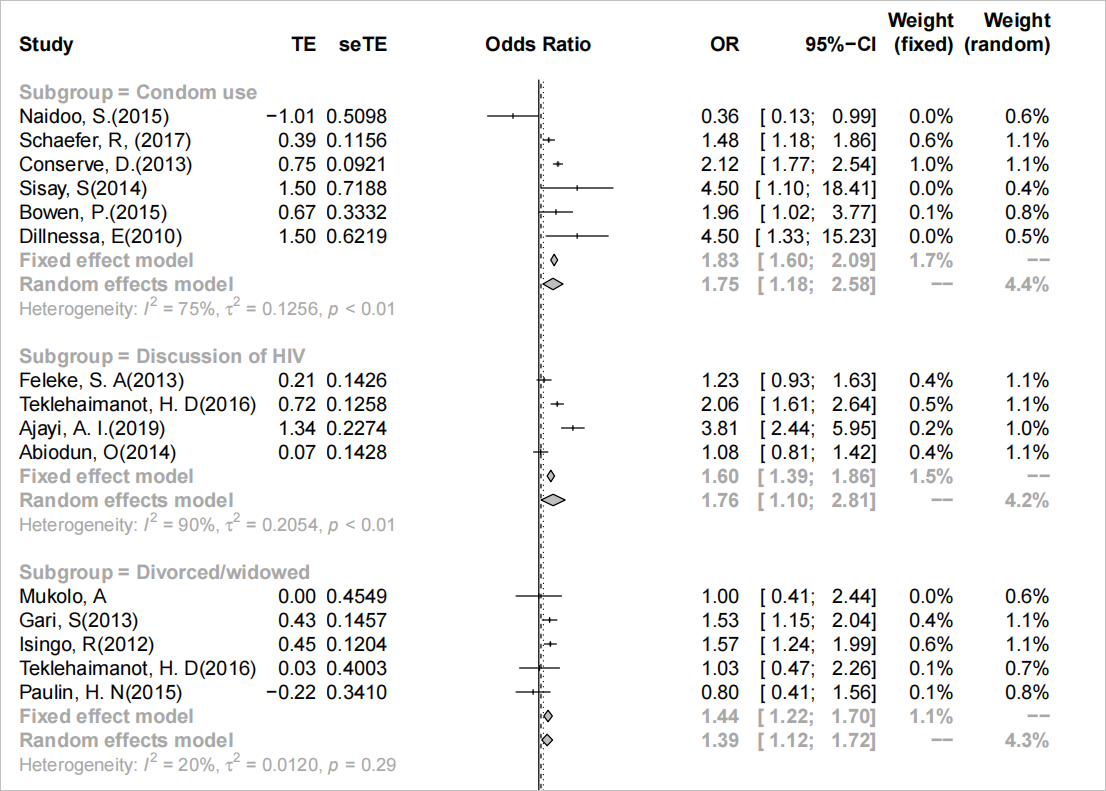


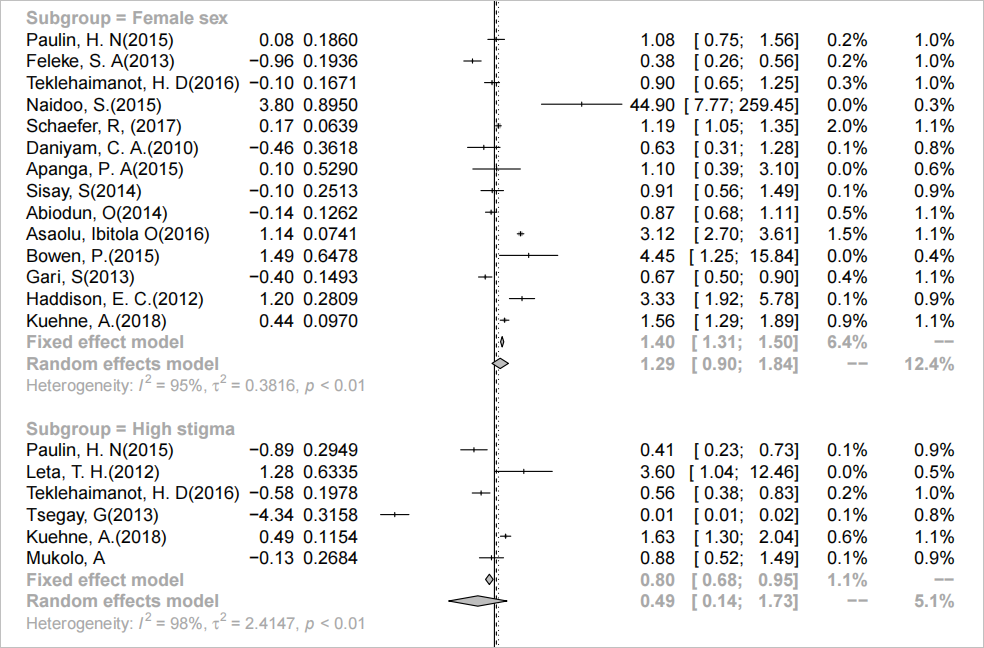


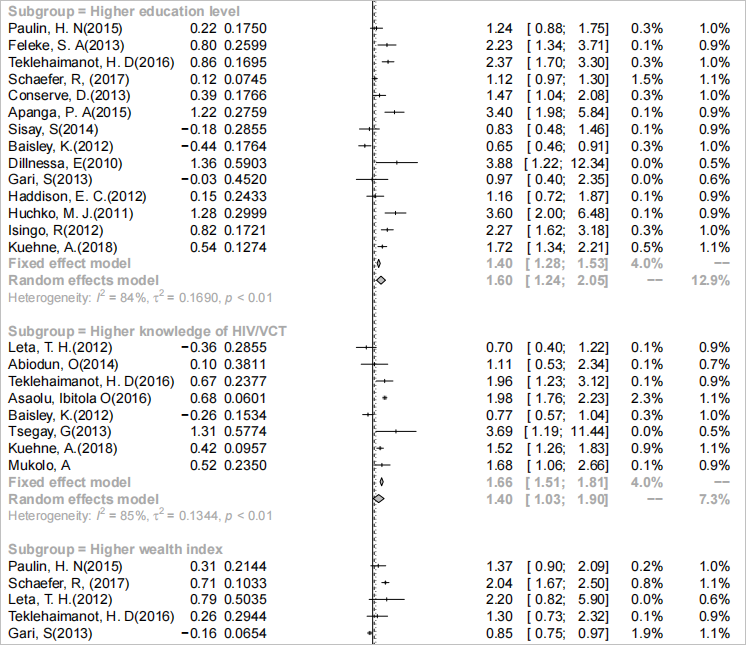


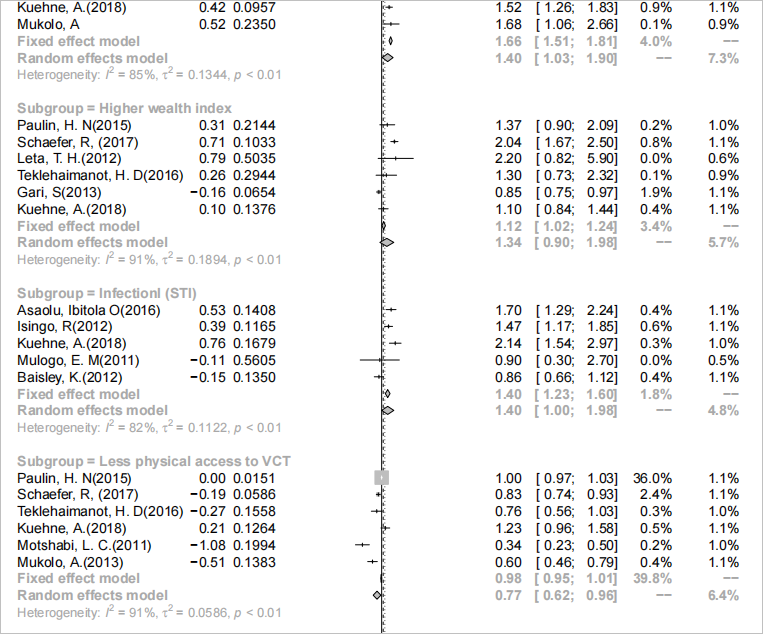


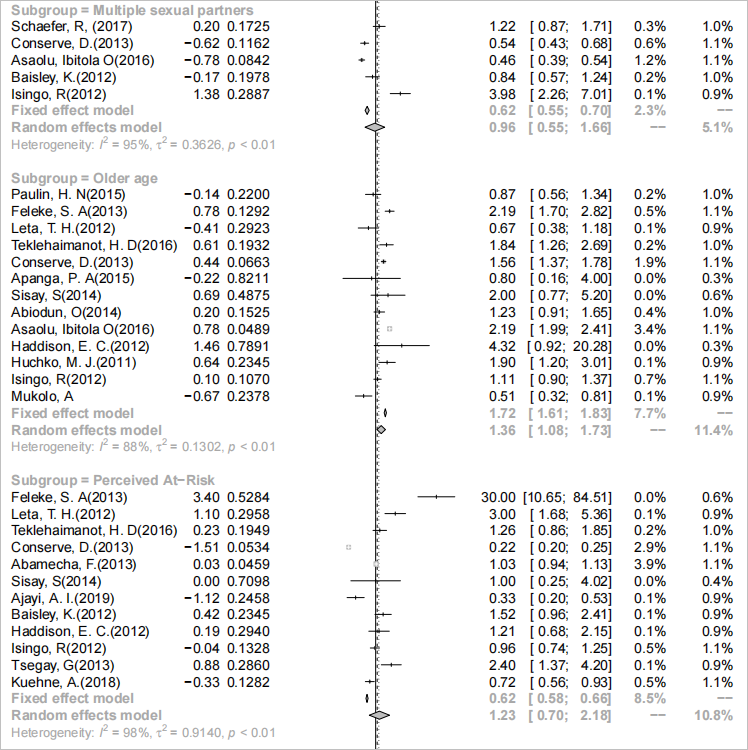


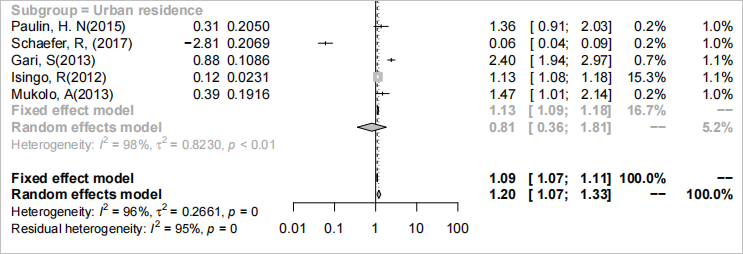


**Table S6. PRISMA checklist for reporting of systematic reviews and meta-analysis**

| **Section/topic** | **#** | **Checklist item** | **Reported on page #** |
| --- | --- | --- | --- |
| **TITLE** | | |  |
| Title | 1 | Identify the report as a systematic review, meta-analysis, or both. | 1 |
| **ABSTRACT** | | |  |
| Structured summary | 2 | Provide a structured summary including, as applicable: background; objectives; data sources; study eligibility criteria, participants, and interventions; study appraisal and synthesis methods; results; limitations; conclusions and implications of key findings; systematic review registration number. | 3 |
| **INTRODUCTION** | | |  |
| Rationale | 3 | Describe the rationale for the review in the context of what is already known. | 4 |
| Objectives | 4 | Provide an explicit statement of questions being addressed with reference to participants, interventions, comparisons, outcomes, and study design (PICOS). | 5 |
| **METHODS** | | |  |
| Protocol and registration | 5 | Indicate if a review protocol exists, if and where it can be accessed (e.g., Web address), and, if available, provide registration information including registration number. | 5 |
| Eligibility criteria | 6 | Specify study characteristics (e.g., PICOS, length of follow-up) and report characteristics (e.g., years considered, language, publication status) used as criteria for eligibility, giving rationale. | 5 |
| Information sources | 7 | Describe all information sources (e.g., databases with dates of coverage, contact with study authors to identify additional studies) in the search and date last searched. | 5 |
| Search | 8 | Present full electronic search strategy for at least one database, including any limits used, such that it could be repeated. | 5 |
| Study selection | 9 | State the process for selecting studies (i.e., screening, eligibility, included in systematic review, and, if applicable, included in the meta-analysis). | 5 |
| Data collection process | 10 | Describe method of data extraction from reports (e.g., piloted forms, independently, in duplicate) and any processes for obtaining and confirming data from investigators. | 5 |
| Data items | 11 | List and define all variables for which data were sought (e.g., PICOS, funding sources) and any assumptions and simplifications made. | 6 |
| Risk of bias in individual studies | 12 | Describe methods used for assessing risk of bias of individual studies (including specification of whether this was done at the study or outcome level), and how this information is to be used in any data synthesis. | 6 |
| Summary measures | 13 | State the principal summary measures (e.g., risk ratio, difference in means). | 6 |
| Synthesis of results | 14 | Describe the methods of handling data and combining results of studies, if done, including measures of consistency (e.g., I^2^) for each meta-analysis. | 6 |
| **Section/topic** | **#** | **Checklist item** | **Reported on page #** |
| Risk of bias across studies | 15 | Specify any assessment of risk of bias that may affect the cumulative evidence (e.g., publication bias, selective reporting within studies). | 7 |
| Additional analyses | 16 | Describe methods of additional analyses (e.g., sensitivity or subgroup analyses, meta-regression), if done, indicating which were pre-specified. | 8 |
| **RESULTS** | | |  |
| Study selection | 17 | Give numbers of studies screened, assessed for eligibility, and included in the review, with reasons for exclusions at each stage, ideally with a flow diagram. | 8 |
| Study characteristics | 18 | For each study, present characteristics for which data were extracted (e.g., study size, PICOS, follow-up period) and provide the citations. | 8 |
| Risk of bias within studies | 19 | Present data on risk of bias of each study and, if available, any outcome level assessment (see item 12). | 9 |
| Results of individual studies | 20 | For all outcomes considered (benefits or harms), present, for each study: (a) simple summary data for each intervention group (b) effect estimates and confidence intervals, ideally with a forest plot. | 9 |
| Synthesis of results | 21 | Present results of each meta-analysis done, including confidence intervals and measures of consistency. | 9 |
| Risk of bias across studies | 22 | Present results of any assessment of risk of bias across studies (see Item 15). | 10 |
| Additional analysis | 23 | Give results of additional analyses, if done (e.g., sensitivity or subgroup analyses, meta-regression [see Item 16]). | 10 |
| **DISCUSSION** | | |  |
| Summary of evidence | 24 | Summarize the main findings including the strength of evidence for each main outcome; consider their relevance to key groups (e.g., healthcare providers, users, and policy makers). | 11 |
| Limitations | 25 | Discuss limitations at study and outcome level (e.g., risk of bias), and at review-level (e.g., incomplete retrieval of identified research, reporting bias). | 10 |
| Conclusions | 26 | Provide a general interpretation of the results in the context of other evidence, and implications for future research. | 13 |
| **FUNDING** | | |  |
| Funding | 27 | Describe sources of funding for the systematic review and other support (e.g., supply of data); role of funders for the systematic review. | 14 |
| *Reference*: Moher D, Liberati A, Tetzlaff J, Altman DG, The PRISMA Group (2009). Preferred Reporting Items for Systematic Reviews and Meta-Analyses: The PRISMA Statement. PLoS Med 6(7): e1000097. doi:10.1371/journal.pmed1000097. | | | |
